# Repurposing Niflumic Acid-Loaded PEGylated Cerosomes for Topical Solid Ehrlich’s Carcinoma Management via EGFR/ERK/miR-21 Signaling Pathway Modulation

**DOI:** 10.3390/pharmaceutics18091125

**Published:** 2026-09-07

**Authors:** Mona M. Mostafa, Shaimaa Mosallam, Mai M. Eltaweel, Maha M. Amin, Jawaher Abdullah Alamoudi, Heba Mohammed Refat M. Selim, Mira Magdy William, Shady M. Abd El-Halim

**Affiliations:** 1 Department of Pharmaceutics and Industrial Pharmacy, Faculty of Pharmacy, October 6 University, 6th of October City, Giza 12585, Egypt; mona.mohammed.ph@o6u.edu.eg (M.M.M.); shaimaamosallam@o6u.edu.eg (S.M.); 2 Postgraduate Researcher, Faculty of Pharmacy, Cairo University, Cairo 11562, Egypt; 3 Department of Pharmaceutics and Industrial Pharmacy, Faculty of Pharmacy, Cairo University, Cairo 11562, Egypt; mai.eltaweel@pharma.cu.edu.eg (M.M.E.); maha.amin@pharma.cu.edu.eg (M.M.A.); 4 Department of Pharmaceutical Sciences, College of Pharmacy, Princess Nourah bint Abdulrahman University, Riyadh 11671, Saudi Arabia; jaalamoudi@pnu.edu.sa; 5 Department of Pharmaceutical Sciences, College of Pharmacy, AlMaarefa University, Diriyah, Riyadh 13713, Saudi Arabia; hmustafa@um.edu.sa; 6 Research Center, Deanship of Scientific Research and Post-Graduate Studies, AlMaarefa University, Diriyah, Riyadh 13713, Saudi Arabia; 7 Department of Biochemistry, Faculty of Pharmacy, October 6 University, 6th of October City, Giza 12585, Egypt; miramagdy@o6u.edu.eg

**Keywords:** repurposing, NIF-loaded PEG-CERs, MAPK-ERK signaling pathway, miR-21-5p modulation, subcutaneous solid Ehrlich carcinoma, topical skin cancer management

## Abstract

**Background/Objectives**: Repurposing existing drugs may represent a promising strategy for effective cancer therapy. This study was the first to investigate the augmented antitumor therapeutic effect achieved by co-incorporating the NSAID Niflumic acid (NIF) with ceramides into PEGylated cerosomes (NIF-loaded PEG-CERs) in a novel platform that targets specifically the MAPK-ERK signaling pathway and miR-21-5p modulation. **Methods**: The prepared formulae were statistically optimized utilizing a full factorial design and the optimal formula (C5) was further incorporated into a topical gel and evaluated for ex vivo rat skin permeation, and tested in vivo in a subcutaneous solid Ehrlich carcinoma (SEC) mice model. **Results**: The optimal formula (C5) showed tubular elongated morphology with higher EE% (96.71 ± 0.0), lower vesicular size (VS) and PDI values, 292.95 ± 0.78 nm and 0.47 ± 0.0 respectively. A high ZP value (−37.5 ± 0.57 mV) was in accordance with stability results showing good stability of the optimal formula (C5). Permeability studies exhibited 2.02-fold higher skin permeation compared to pure NIF gel. A significant decrease in tumor volume and marked improvement in survival rate in SEC mice were confirmed by downregulation of EGFR, ERK1, ERK2, and miR-21-5p expression. Furthermore, an increase in total antioxidant capacity and caspase-3 levels was observed, accompanied by significant suppression in cyclin D1, MMP-2, COX-2, and MDA levels. Finally, histopathological analysis revealed the superior antitumor effect of C5 gel together with immunohistochemical assay showing the lowest BCL-2-positive staining, indicating the restoration of physiological apoptotic balance. **Conclusions**: Based on the previous findings, NIF-loaded PEG-CERs offer augmented therapeutic potential for efficient topical skin cancer management in an SEC mice model.

## 1. Introduction

Skin cancers represent are significantly prevalent among cancer types globally, with their incidence consistently increasing over the past few decades [1,2,3,4]. Factors contributing to skin carcinogenesis encompass chronic cutaneous inflammation, ultraviolet radiation (UVA and UVB), viral infections, and various inflammation-inducing agents and traumas [5,6].

Despite advancements in early detection and treatment, managing skin cancer remains a significant challenge [2]. Traditional therapies commonly employed in the treatment of skin cancer have several limitations; therefore, there is a significant demand for the creation of novel anti-cancer therapeutics capable of overcoming these challenges [7,8,9].

Skin cancer cells rely on cell-signaling pathways for their proliferation, metabolic support, survival, and development. Critically, alterations in these mechanisms can trigger the development of cancerous tissue growth [10]. The mitogen-activated protein kinase (MAPK)/extracellular signal-regulated kinase 1/2 (ERK) pathway (Ras/Raf/MEK/ERK) is a crucial signaling cascade that regulates skin cell survival, growth, and differentiation. It functions by transmitting extracellular signals from the cell membrane to the nucleus via a cascade of phosphorylation processes. However, its hyperactivation drives most skin cancers, typically through mutations in BRAF or RAS, along with other genetic or epigenetic alterations, resulting in uncontrolled proliferation and therapeutic resistance. Such dysregulation ultimately promotes cell survival, migration, invasion, metastasis, and angiogenesis [11,12,13,14].

It is particularly important to note that extracellular signals, such as cytokines or chemokines, secreted in the inflammatory microenvironment, could trigger the MAPK pathway through binding to a cytokine receptor tyrosine kinase, thereby activating tyrosine kinases like Janus Kinase-3 (Jak-3) [15]. In view of these mechanisms, repurposing non-oncology drugs for cancer therapy presents a potentially attractive, cost-effective, and rapid alternative to traditional drug development [9,16].

Drug repurposing, also known as reprofiling, involves identifying novel applications for existing licensed and FDA-approved medications, therefore avoiding the elaborate processes and high costs associated with conventional drug development. Drug repurposing for cancer therapy has recently garnered attention as a strategy to address well-established drug resistance challenges and to tailor treatment plans that reduce adverse side effects for patients [9,16,17].

Niflumic acid (NIF), a selective cyclooxygenase-2 (COX-2) inhibitor, is a member of the non-steroidal anti-inflammatory drugs (NSAIDs) group and a derivative of anthranilic acid. It is traditionally utilized to treat inflammatory pain, fever, and rheumatoid diseases [18,19,20]. Along with its anti-inflammatory activity, NIF has been noted to inhibit cell proliferation and migration in a few cancer cell line studies [21,22]. However, the oral use of NIF induces multiple adverse effects common to NSAIDs, including gastrointestinal irritation and ulceration, agranulocytosis, renal insufficiency, hepatotoxicity, nausea, vomiting, cutaneous reactions, and neutropenia [23,24]. In addition, NIF suffers from low bioavailability, which is related to NIF’s inadequate aqueous solubility and substantial first-pass metabolism [23].

To overcome these limitations, the topical route represents a more effective approach for treating skin cancer [2]. Nevertheless, penetrating the skin barrier remains a significant challenge in topical delivery [2,24]. Advancements in topical drug delivery have recently accelerated research efforts targeted at the chemopreventive and therapeutic management of skin malignancies [1]. Various nanocarrier systems have been shown to inhibit the inception of skin malignancies at their underlying causes [1,25]. For instance, dysfunctional sphingolipid metabolism is increasingly recognized as a marker of oncogenesis [26,27].

Cerosomes (CERs) are ceramide-encapsulated tubular nano-vesicles prepared by utilizing various surfactants and phospholipids. They demonstrate excellent skin permeability, tolerability, and substantial drug bioavailability upon topical application [28]. Ceramides belong to the group of sphingolipids, which constitute a major component of biological membranes (accounting for approximately 50% of the lipid content in human skin). They are essential for maintaining skin barrier function and play significant roles in several biological processes, which include cell proliferation, cell differentiation, and cell apoptosis [29,30,31].

Ceramides also contribute structurally to cell membranes via enhancing rigidity, forming micro-domains (the rafts and caveolae), along with modulating membrane permeability, each of which is integral to cell signaling [30]. Hence, incorporating ceramides in topical formulations as crucial lipids seems to modify skin barrier properties [30]. Additionally, short-chain ceramides exhibit selective cytotoxicity, inducing cell death in multiple neoplastic cell lines, which include melanoma, breast cancer, pancreatic cancer, and hepatocellular carcinoma [26,27]. Mechanistically, ceramide promotes the dephosphorylation of pro-mitogenic signaling pathways, like ERK, AKT, and signal transducer and activator of transcription-3 (STAT-3) [26,32]. Based on these characteristics, three ceramide molecules belonging to the phytosphingosine class (ceramide VI, III, and IIIB) were selected for use in this study.

However, ceramides’ limited aqueous solubility has impeded their application in cancer therapy [27,33]. Thus, the inclusion of surfactants in cerosomal formulations improves vesicular stability and extends their residence time, resulting in enhanced drug efficacy [9]. Brij surfactants have garnered notable interest owing to their potential applications, including stabilizers within drug-release systems, biodegradable enhancers, and constituents in micellar-catalyzed systems and pH-responsive nanoparticles [34]. In light of these characteristics, two Brij derivatives were utilized in this study to stabilize the prepared CERs.

Supporting the development of an innovative and effective therapeutic strategy, the novelty of the present work lies in achieving an augmented therapeutic effect through the incorporation of the repurposed anti-inflammatory agent (NIF) within diverse PEGylated cerosomes (NIF-loaded PEG-CERs). The present study hypothesized that this incorporation (NIF-loaded PEG-CERs) would enhance its skin delivery and antitumor activity compared with NIF alone. We further hypothesized that the optimized NIF-loaded PEG-CERs gel would exert enhanced antitumor effects through modulation of the MAPK/ERK pathway and miR-21-5p, accompanied by alterations in apoptosis, inflammation, and oxidative stress proposing novel potential mechanisms for skin cancer treatment.

Accordingly, this study aimed to develop and optimize NIF-loaded PEG-CERs, evaluate the ex vivo skin permeability of the optimized formulation, and investigate its in vivo antitumor efficacy and associated molecular, histopathological, and immunohistochemical changes in a subcutaneous solid Ehrlich carcinoma (SEC) model.

## 2. Materials and Methods

### 2.1. Materials

NIF was purchased from Pharo Pharma for Pharmaceuticals (Alexandria, Egypt). Ceramides (VI, III, and IIIB) were kindly provided as a gift from Evonik Industries AG (Essen, Germany). L-α-phosphatidylcholine (derived from egg yolk, ~60% by TLC) was obtained from Sigma Aldrich Chemical Co. (St. Louis, MO, USA). Brij 35 and Brij 93 were obtained from Merck-Schuchardt (Hohenbrunn, Germany). Chloroform, methanol, potassium chloride, potassium dihydrogen phosphate, disodium hydrogen phosphate, and sodium chloride were obtained from El-Nasr Pharmaceutical Chemicals Co. (Cairo, Egypt).

### 2.2. Experimental Design

A full (2^3^.3^1^) factorial design was developed to assess the impact of four independent variables on the PEGylated cerosomal formulations (PEG-CERs). One variable, ceramide type (X1), was set at three levels, while the remaining variables, ceramide amount (X2), Brij type (X3), and sonication time (X4), were studied at two levels each. Entrapment efficiency percentage (EE%; Y1), vesicle size (VS; Y2), polydispersity index (PDI; Y3), and zeta potential (ZP; Y4) were chosen as the dependent variables to reflect vesicular characteristics, as shown in Table 1. All data were analyzed through one-way analysis of variance (ANOVA), establishing a significance level at (*p* < 0.05), utilizing Design Expert^®^ software (version 7; Stat-Ease, Inc., Minneapolis, MN, USA) to determine the optimal formula.

### 2.3. Preparation of NIF-Loaded PEG-CERs

PEGylated cerosomes (PEG-CERs) were prepared utilizing the thin-film hydration technique [9]. A total of 24 formulae, encompassing each possible combination of the studied factors, were prepared in accordance with the experimental design. Briefly, NIF (10 mg), L-α-phosphatidylcholine (100 mg), together with variable amounts and types of ceramides and two different derivatives of Brij, as shown in Table 2, were accurately weighed and dissolved in chloroform (10 mL) within a 250 mL round-bottom flask. Then, the organic solvent (chloroform) was evaporated under reduced pressure utilizing a rotary evaporator (Rotavapor, Heidolph VV 2000, Burladingen, Germany), which rotated at 60 °C and 150 rpm until a dry lipidic film formed on the interior surface of the flask.

Subsequently, the formed film was thoroughly hydrated for 1 h, utilizing 10 mL of deionized water under normal pressure at 60 °C, a temperature exceeding the lipid phase transition temperature [28]. Glass beads were added during the hydration step to boost film hydration efficiency [35]. To evaluate the impact of sonication on the characteristics of the prepared PEG-CERs, the formulated PEG-CERs were subjected to sonication for 10 min utilizing an ultrasonic bath (Model SH 150-41; MTI Corporation, Richmond, CA, USA), operating at a power output of 150 W and a frequency of 40 kHz, in accordance with the study design. To strictly prevent localized heating and heat accumulation, the sample container was immersed in an ice bath. The temperature was continuously monitored to ensure it was maintained at a relatively low temperature throughout the 10-min cycle. Finally, the vesicles’ dispersion was stored in a refrigerator overnight to allow vesicle maturation.

### 2.4. In Vitro Characterization of NIF-Loaded PEG-CERs

#### 2.4.1. Determination of the EE%

The percentage of NIF encapsulated in PEG-CERs was indirectly assessed using a cooling ultracentrifuge (Sigma 3–30 KS, Sigma Laborzentrifugen GmbH, Osterode am Harz, Germany) operated at 20,000 rpm and 4 °C for one hour. The amount of unentrapped NIF was quantified using an ultraviolet (UV) spectrophotometer (Shimadzu, model UV-1601 PC, Kyoto, Japan) to determine the wavelength at 289 nm. The EE% of NIF was determined utilizing the equation declared by Abdelbari et al. [24].

#### 2.4.2. Determination of the Mean VS, PDI, and ZP

The VS, PDI, and ZP of the formed PEG-CERs were assessed after proper dilution using deionized water, utilizing the ZetaSizer (Malvern Panalytical, Malvern, UK) [36,37]. All measurements were performed in triplicate.

### 2.5. Optimization of NIF-Loaded PEGylated Cerosomes

According to a desirability function, the optimum PEG-CERs formula was elected based on achieving the highest EE%, the lowest VS values, while maintaining the PDI and ZP values within acceptable ranges. This approach enabled the simultaneous evaluation of all constraints and responses, facilitating the selection of the formula having the highest desirability value. The adjusted and predicted responses were compared to validate the accuracy of the model performance [35]. The optimized formula was subsequently subjected to further characterization studies.

### 2.6. Characterization of the Selected NIF-Loaded PEG-CERs Formula

#### 2.6.1. Transmission Electron Microscope (TEM)

The morphology of the optimal NIF-loaded PEG-CERs and its corresponding formula that subjected to sonication for 10 min, was analyzed using a transmission electron microscope (TEM) (JEOL JEM 2100, Tokyo, Japan), according to the method described by Albash et al. [28].

#### 2.6.2. Differential Scanning Calorimetry (DSC)

A differential scanning calorimeter (Shimadzu-DSC 60, Kyoto, Japan) was utilized for DSC analysis on pure NIF and the lyophilized optimum NIF-loaded PEG-CERs formula (freeze dryer; Christ, Osterode am Harz, Germany, Alpha 1-2LD plus), as outlined by Lamie et al. [38].

#### 2.6.3. Fourier-Transform Infrared Spectroscopy (FTIR)

FTIR spectroscopy was used as the primary verification technique to assess structural compatibility between NIF and the excipients and to confirm the successful encapsulation of the drug within the final PEG-CERs system. The spectral profiles of the pure NIF, the optimum blank (drug-free) vesicles, a physical mixture of NIF with the optimum PEG-CERs excipients, and the lyophilized optimum NIF-loaded PEG-CERs formulation were obtained utilizing an IRAFFINITY-1 FTIR device (Shimadzu, Kyoto, Japan), as described by Soliman et al. [39].

### 2.7. Short-Term Physical Stability Study

The physical stability of the optimum PEG-CERs was examined to assess the extent of drug leakage, vesicle growth, or any other physical alterations. The optimized formula was kept in a refrigerator at 5 ± 3 °C for a duration of three months, and its stability was assessed by determining the EE%, VS, PDI, and ZP after 45 and 90 days, in comparison with the initial measurements of the freshly prepared formulae. Results were analyzed statistically utilizing Student’s t-test through SPSS^®^ software (version 22.0). Furthermore, the system was examined visually for any signs of vesicle sedimentation or aggregation [40,41].

### 2.8. Formulation of the Optimal NIF-Loaded PEG-CERs Gel

In this study, we picked gel as the final skin product to enhance the retention of the optimized PEG-CERs formula and improve topical application. This was achieved by incorporating 1% w/w hydroxypropyl methylcellulose (HPMC) into the optimal NIF-loaded PEG-CERs formula, blank gel formulation and the NIF suspension, using a magnetic stirrer to facilitate gel formation [24]. HPMC is employed as a gelling agent due to its minimal toxicity, ability to produce a clear gel, and ease of dissolving in water. Furthermore, HPMC produced a clear, neutral, and colorless gel that is stable within the pH range of 3–11, providing robust resistance to microbial growth and enhances the strength of the film upon drying on the skin [42].

Solid tumors exhibit a mildly acidic extracellular microenvironment, with extracellular pH values typically ranging from approximately 6.5 to 6.9, resulting from altered tumor metabolism and heightened acid production [43]. Accordingly, the pH of the gel formulations was adjusted to 6.83 ± 0.07, which falls within the reported range of the tumor extracellular microenvironment. This agrees with a study conducted by Lamie et al. [38] in which they prepared itraconazole aspasomal cream (with a pH value of 6.84 ± 0.21) in treating subcutaneous Ehrlich carcinoma as a skin cancer model.

### 2.9. Ex Vivo Skin Permeation Study

The permeation experiment was conducted on the optimal NIF-loaded PEG-CER gel (1 g) and the corresponding NIF gel (1 g), each equivalent to 1 mg NIF. The study was performed on excised rat skin using cylindrical diffusion tubes (plastic tubes open at both ends) with an effective diffusion area of 1.77 cm^2^, corresponding to a circular surface with a radius of 0.885 cm. Each tube was sealed at one end with the skin membrane, ensuring that the stratum corneum faced the donor compartment, while the other end was attached to the USP dissolution apparatus II (Distek, Model 2500, New Jersey, USA). A 100 mL of phosphate-buffered saline (PBS) solution (pH 7.4) was employed as the permeation medium and kept at 37 °C ± 0.5 °C under continuous stirring at 100 rpm while maintaining sink conditions. Samples were collected at predetermined time intervals over 24 h, and immediately an equal volume of fresh medium was added. The samples were analyzed spectrophotometrically at a wavelength of 289 nm [16,24].

The permeation profile for NIF was established by plotting the cumulative quantity of the drug permeated (Q) per unit area against time. Steady state flux (J_ss_, µg/cm^2^.h), permeability coefficient (*P*_app_, cm/h), and the enhancement factor were calculated in accordance with Latif et al. [44].

The leaching of endogenous, UV-absorbing skin constituents into the receptor medium during the 24 h permeation study could potentially interfere with the UV-based quantification of NIF if not properly accounted for. Accordingly, a blank-skin control was included in the ex vivo permeation study. An identical piece of untreated rat skin was mounted in the diffusion cell, exposed to PBS (pH 7.4) under identical experimental conditions (37 °C for 24 h), and sampled in the same manner without applying any drug-loaded formulation (neither the free NIF gel nor the optimum C5 formulation). The receptor medium collected from the blank-skin control was analyzed under the same conditions as the test samples. This accounted for any endogenous UV-absorbing substances released from the skin during the diffusion experiment, thereby confirming that the measured absorbance was attributable solely to the investigated drug rather than biological interfering components.

### 2.10. In Vivo Evaluation of the Anticancer Efficacy of the Optimum NIF-Loaded PEG-CERs Gel

The current in vivo study was conducted in accordance with the guidelines of care and use of laboratory animals approved by Research Ethical Committee, Faculty of Pharmacy, Cairo University, Egypt, (REC-FOPCU); PI (3807).

#### 2.10.1. Animals

Fifty adult male Swiss Albino mice, each weighing between 20 and 25 g, were obtained from the National Research Center in Cairo, Egypt. Mice were randomized, housed, and maintained for one-week acclimatization period under atmospheric conditions of 25 ± 1 °C, 50% relative humidity in a dark/light cycle for 12 h. They had unrestricted access to a standard chow diet and water throughout the study.

#### 2.10.2. Tumor Induction

Ehrlich Ascites Carcinoma (EAC) cells, at a concentration of 1 × 10^6^ cells, were acquired from the Pharmacology and Experimental Oncology Unit of the National Cancer Institute, located at Cairo University in Giza, Egypt. EAC cells were implanted into the peritoneal cavity of a mouse and permitted to proliferate. Within 8–10 days, ascitic fluid that contained Ehrlich tumor cells was obtained, collected utilizing a sterile syringe, then diluted in 0.9% sterile saline (1:9 *v*/*v*), and counted using a Neubauer Hemocytometer (Sigma Aldrich, St. Louis, MO, USA). To produce a subcutaneous solid Ehrlich carcinoma (SEC), viable EAC cells (2.5 × 10^6^ cells) were subcutaneously injected into the upper dorsal side of the right hind leg of mice. Following the formation of a palpable solid tumor mass (about 300 mm^3^) within 12 days, the hair over the tumor site was removed, and topical application of the different treatments was initiated.

Consequently, the animals were randomly assigned to five groups (*n* = 10 mice per group) as follows: Group I (negative control) consisted of normal, non-tumor-bearing mice; Group II (SEC control) consisted of tumor-bearing mice that were left untreated after subcutaneous tumor induction and received only food and water; Group III (SEC + NIF gel), tumor-bearing mice were topically treated with 1 g of NIF gel (equivalent to 1 mg NIF) twice daily [24]; Group IV (SEC + blank gel formulation), tumor-bearing mice treated topically with 1 g of the optimal blank PEG-CERs gel formulation twice daily; and Group V (SEC + optimal NIF-loaded gel formulation), tumor-bearing mice treated topically with 1 g of the optimal NIF-loaded PEG-CERs gel formulation twice daily. The treatment duration was 21 days.

During the treatment period, the dimensions of the tumor were measured with a vernier caliper (Tricle Brand, Shanghai, China) starting from the 12th day post-inoculation (day 0) and subsequently, every 4–5 days till the end of the study. The tumors’ volume was determined utilizing the following formula: tumor volume (mm^3^) = 0.52 × Length × Width^2^ [45]. The tumor inhibition rate (TIR) was evaluated utilizing the following formula: TIR = (mean tumor volume of the control tumor group − mean tumor volume of the treated group) × 100/mean tumor volume of the control tumor group) [46]. The survival rate of each experimental group was determined by applying the following formula: survival rate = (number of surviving animals in a group on day 21/total number of animals in the same group at the start of the experiment) × 100 [47]. At the end of the study, mice were anesthetized using ketamine (75–100 mg/kg) [48] prior to being sacrificed via cervical dislocation.

#### 2.10.3. Assessment of Biochemical Parameters

The tumor tissue was extracted and partitioned into portions. One portion of the sample was stored at −80 °C till RNA was isolated for subsequent RT-qPCR analysis of epidermal growth factor receptor (EGFR), extracellular signal-regulated kinase 1 (ERK1), extracellular signal-regulated kinase 2 (ERK2), and miR-21-5p. The second portion was homogenized in PBS (ice-cold, pH 7.2–7.4), segregated into multiple aliquots, and stored at −80 °C. One aliquot was centrifuged for 5 min at 5000× *g* and 4 °C for determination of COX-2 and matrix metalloproteinase-2 (MMP-2) levels applying enzyme-linked immunosorbent assay (ELISA) kits (Cat#: MBS160358, MyBioSource, san diego, CA, USA, and Cat#: CSB-E04676m CUSABIO Technology LLC, Wuhan, China, respectively).

Another tumor homogenate aliquot was subjected to centrifugation for 10 min at 10,000× *g* and 4 °C to assess caspase-3 levels using ELISA kit (Cat#: SEA626Mu, Cloud-Clone Corp., Katy, TX, USA) and for colorimetric determination of total antioxidant capacity (TAC) using kit purchased from Assay Genie Ltd. (Dublin, Ireland, Cat#: MAES0147). The last aliquot was subjected to centrifugation for 15 min at 5000× *g* and 4 °C for determination of cyclin D1 levels using ELISA kit (Cat#: MBS727668, MyBioSource, CA, USA) and for colorimetric determination of malondialdehyde (MDA) using kits purchased from Bio-Diagnostic (Cairo, Egypt, Cat#: MD 25 29). All kits were used in accordance with the manufacturers’ directions.

#### 2.10.4. Real-Time Polymerase Chain Reaction (RT-PCR) for EGFR, ERK 1, ERK 2, and miR-21-5p

Utilizing the Direct-zol RNA Miniprep Plus (Cat# R2072, ZYMO Research Corp., Irvine, CA, USA), total RNA was extracted from tumor tissue in accordance with the manufacturer’s protocol and kept at −80 °C for subsequent analysis. The Nanodrop 2000 spectrophotometer (NanoDrop Technologies, Wilmington, DE, USA) was utilized to assess RNA concentration with purity. SuperScript IV Reverse Transcriptase kit (Cat# 18090200, Thermo Fisher Scientific, Waltham, MA, USA) was utilized for reverse transcription of total RNA into its complementary DNA. cDNA was employed for quantitative real-time PCR amplification and analysis through SYBR Green (SYBR^®^ Premix Ex Taq™ II, TaKaRa, Dalian, China), deploying an Applied Biosystem (StepOne™, Foster City, CA, USA). The RT-PCR protocol consisted of an initial denaturation step at 95 °C for 2 min, followed by 40 cycles for 10sec at 95 °C, then at their respective annealing temperatures for 10 s, and an extension for 30 s at 72 °C, followed by a final extension step for 5 min at 72 °C [49]. The expression of GAPDH functioned as the internal control, and the relative expression was defined via 2^−∆∆Ct^ determined [50]. The primer sequences of tested EGFR, ERK 1, ERK 2 and miR-21-5p as well as GAPDH are displayed in Table 3.

#### 2.10.5. Histopathological Study

Following euthanasia, specimens of excised tumors from different groups were fixed in 10% neutral buffered formalin for 48 h and then transferred to 70% ethanol until further processing. Tissue sections were prepared using paraffin embedding, and histopathological sections (5 µm thickness) were stained with hematoxylin and eosin (H&E). The stained sections were examined under a light microscope (Leica DM4B digital microscope accompanied by Leica DMC4500 camera (Leica, Wetzlar, Germany).

#### 2.10.6. Immunohistochemistry ((B-Cell Lymphoma 2 (BCL-2) Expression)

Paraffin-embedded tissue sections were affixed to positively charged slides utilizing the avidin-biotin-peroxidase complex (ABC) method and underwent IHC staining with rabbit anti-BCL-2 antibodies (dil 1:600; Cat# GB114830, Servicebio, Wuhan, China). The staining protocols for the anti- BCL-2 products were carried out in accordance with the manufacturer’s instructions. Sections from each group were incubated with these antibodies, followed by the addition of reagents necessary for the ABC method (Vectastain ABC-HRP Kit; Vector Laboratories, Newark, CA, USA).

Marker expression was labeled with peroxidase and stained with diaminobenzidine (DAB, produced by Sigma) to detect the antigen–antibody complex. All slide-processing procedures included both positive and negative controls. Negative controls included utilizing non-immune serum in place of the primary or secondary antibodies. IHC-stained sections were visualized utilizing an Olympus microscope (BX-63), and the positive expression was quantified as area percentage. Scoring of immunohistochemistry results was quantitatively assessed by determining the percentage of immunopositive reaction area in 10 randomly selected microscopic fields using ImageJ software (version 1.53t; Wayne Rasband and contributors, National Institutes of Health, Bethesda, MD, USA).

#### 2.10.7. Statistical Analysis of Data

The significant variations between the studied formulas’ outcomes were assessed using one-way ANOVA. In addition, an ANOVA followed by Tukey–Kramer multiple comparisons tests was employed to assess variation in the in vivo study. The significance level was set at 0.05; thus, outcomes with *p* < 0.05 were considered statistically significant. The statistical evaluations were conducted using GraphPad Prism software (version 8, San Diego, CA, USA).

## 3. Results and Discussion

### 3.1. Factorial Design Optimization

**Figure 1:**
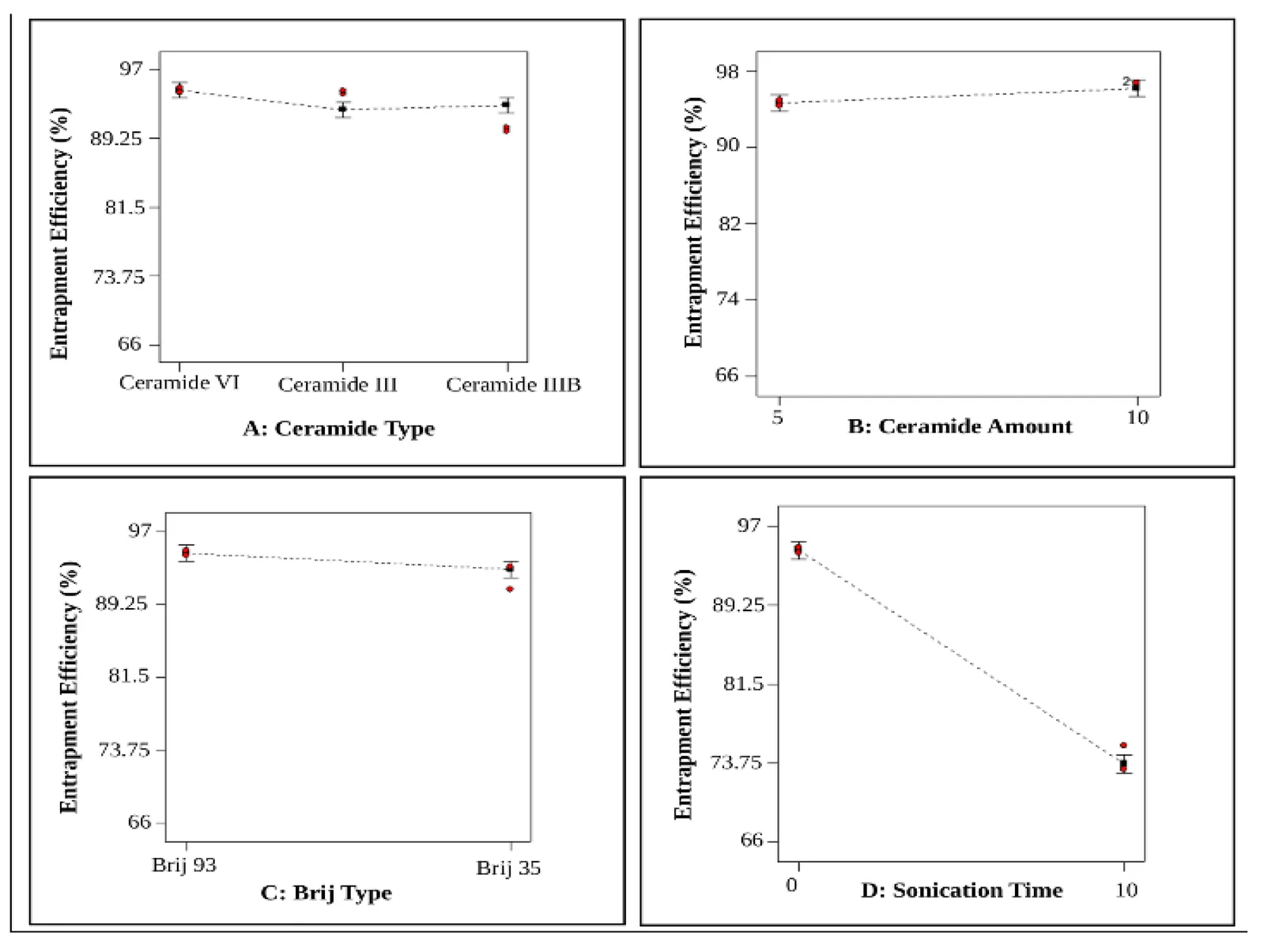
The effect of (**A**) ceramide type, (**B**) Ceramide Amount, (**C**) Brij Type and (**D**) Sonication Time on EE% of NIF-loaded PEG−CERs.

**Figure 2:**
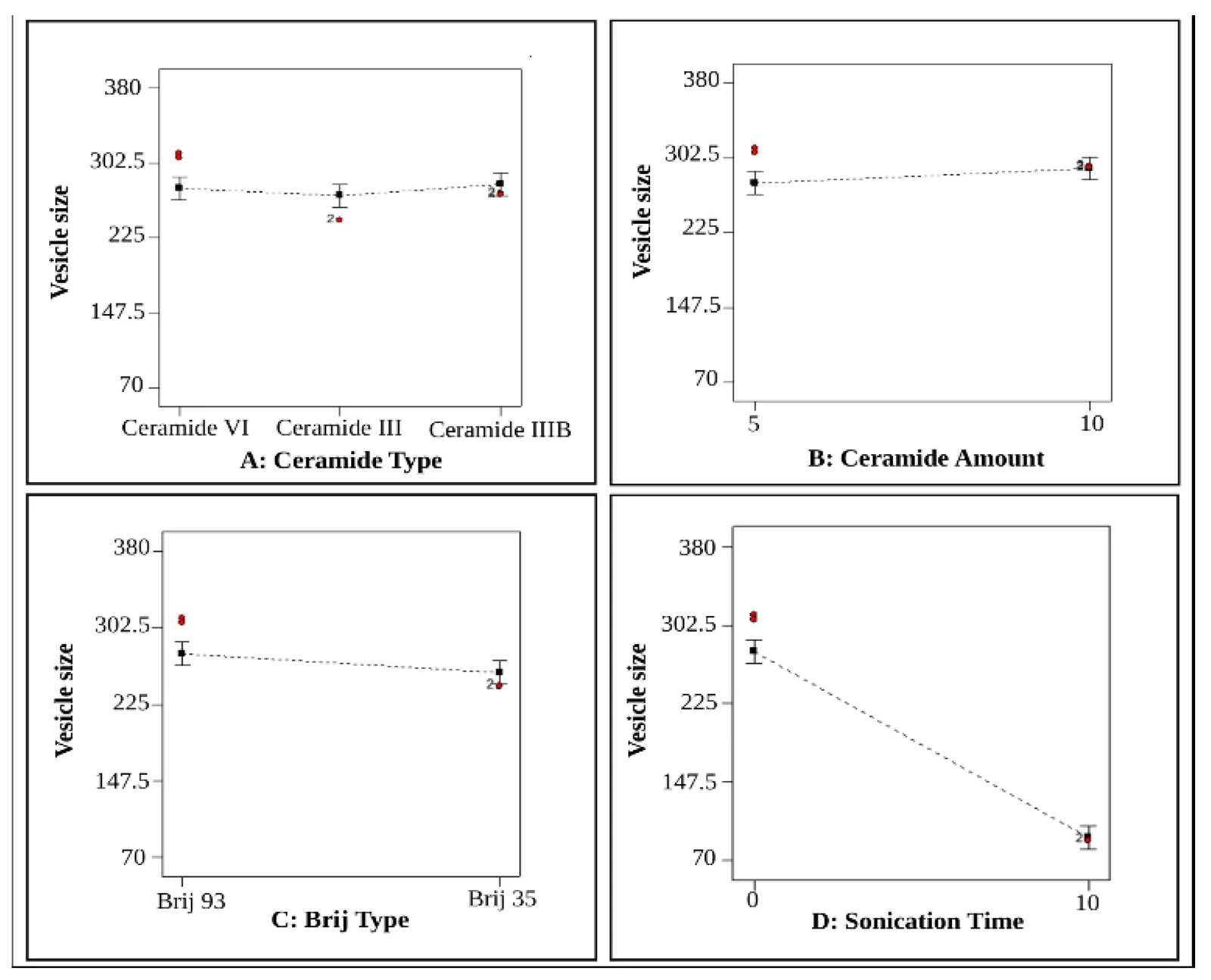
The effect of (**A**) ceramide type, (**B**) Ceramide Amount, (**C**) Brij Type and (**D**) Sonication Time on PS of NIF-loaded PEG−CERs.

**Figure 3:**
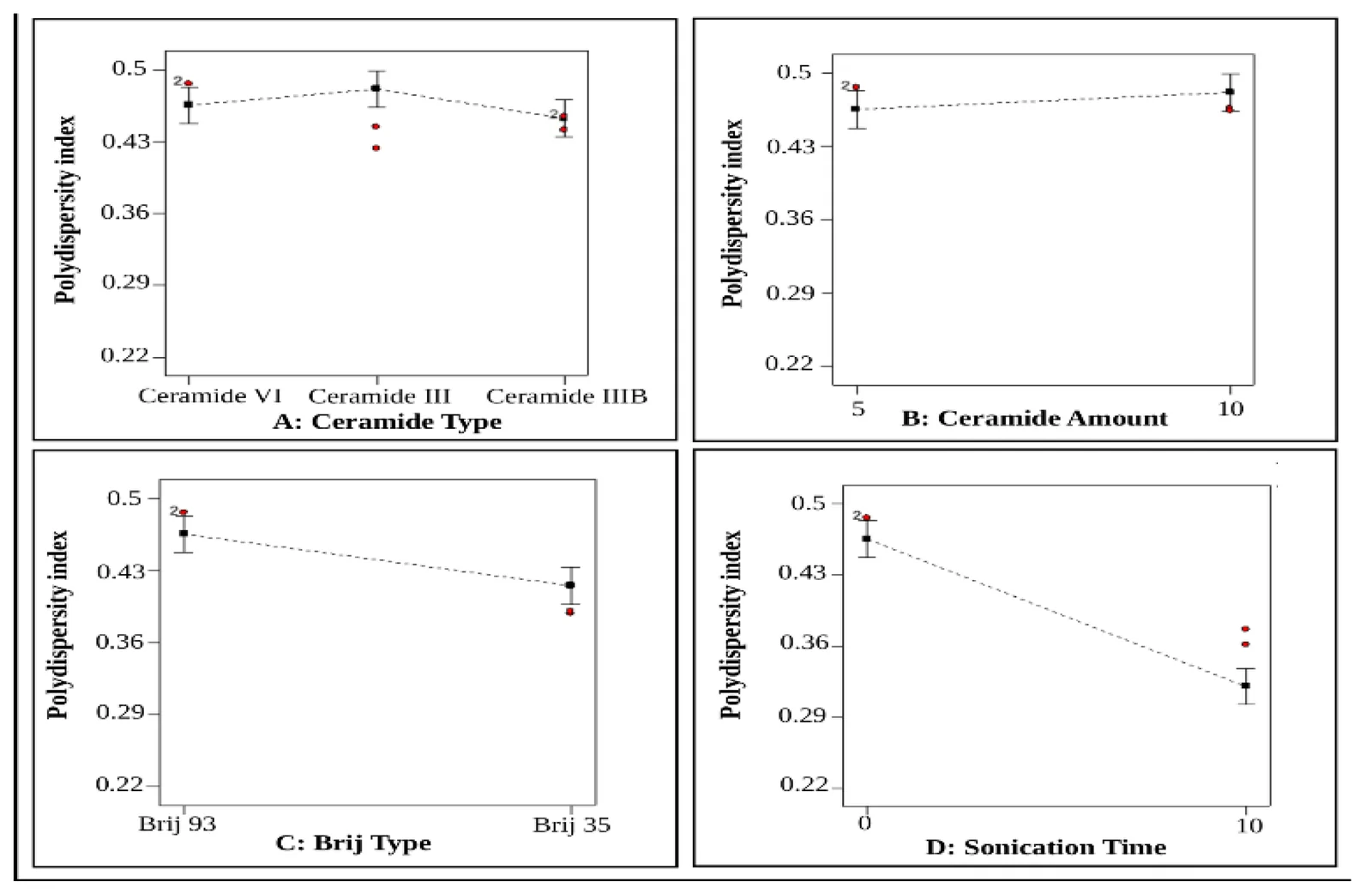
The effect of (**A**) ceramide type, (**B**) Ceramide Amount, (**C**) Brij Type and (**D**) Sonication Time on PDI of NIF-loaded PEG−CERs.

**Figure 4:**
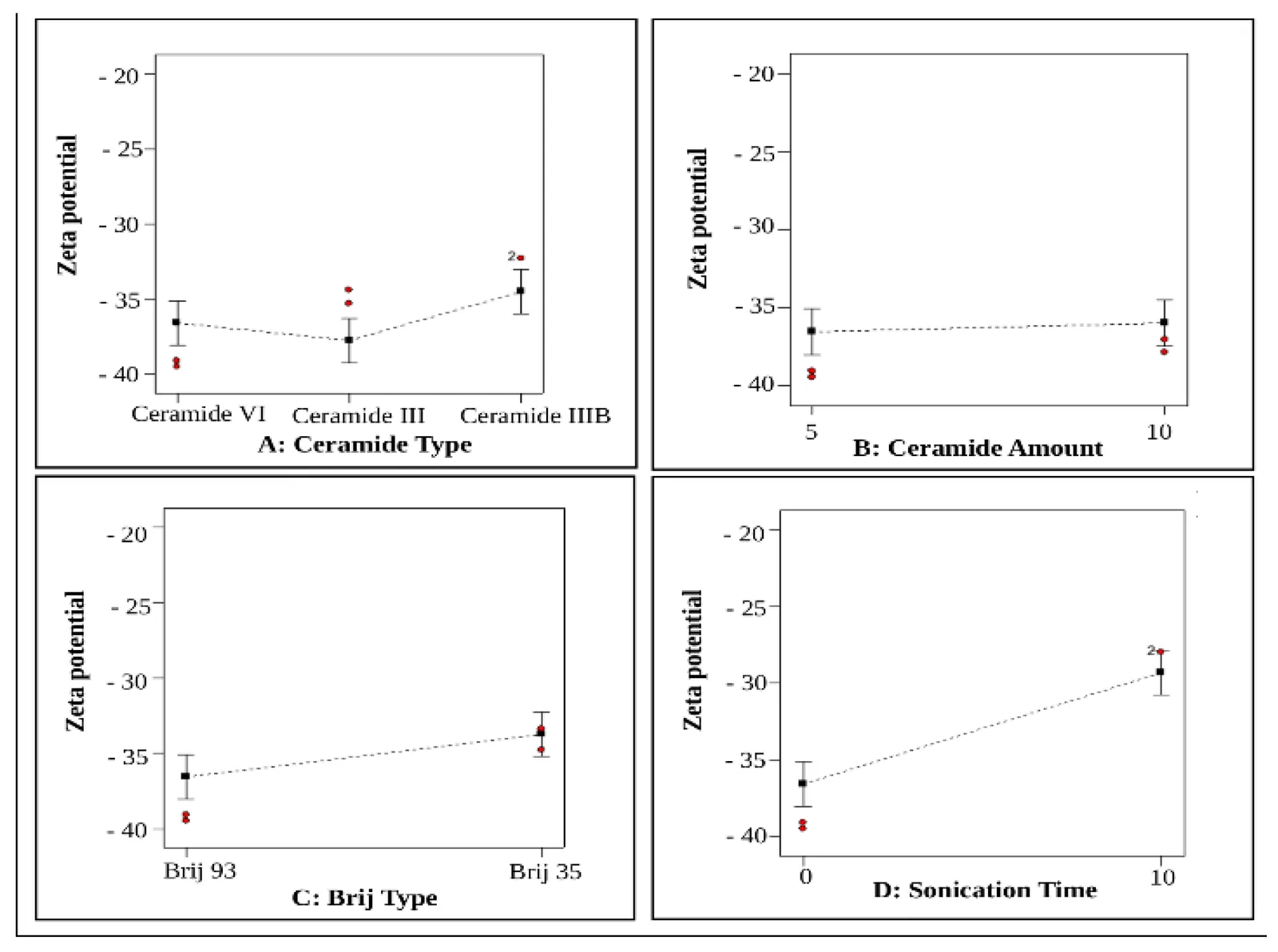
The effect of (**A**) ceramide type, (**B**) Ceramide Amount, (**C**) Brij Type and (**D**) Sonication Time on ZP of NIF-loaded PEG−CERs.

Based on the results of the experimental design analysis, the predicted R^2^ values were found to be in good agreement with the corresponding adjusted R^2^ values across all responses. An adequate precision ratio exceeding four is considered indicative of a desirable signal-to-noise ratio, and this criterion was satisfied for all investigated responses, as presented in Table 4. The effects of the different independent variables, namely ceramide type (X1), ceramide amount (X2), Brij type (X3), and sonication time (X4), are depicted in Figure 1, Figure 2, Figure 3 and Figure 4.

### 3.2. Effect of Formulation Variables on EE% (Y1)

The capability of the developed vesicles to encapsulate a significant amount of drug is a critical parameter for their potential use as a topical drug delivery system. Our study demonstrated that the existence of hydrophobic ceramide and phosphatidylcholine facilitated the significant integration of the water-insoluble NIF molecules within the developed PEG-CERs. This aligns with the findings of other authors [28,51]. Albash and colleagues [28] reported a significant incorporation of water-insoluble Fenticonazole nitrate molecules into the prepared PEG-CERs. Similarly, Abdelgawad et al. [51] showed a notable EE% of a retinoid drug (Tazarotene) in the prepared CERs for clinical application in the treatment of psoriasis. The significance of the independent variables on the NIF EE% is presented in Table 4.

As shown in Table 2, the EE% of the formulated NIF-loaded PEG-CERs ranged from 67.71 ± 1.42% to 96.71 ± 0.0%. ANOVA statistical analysis of the data indicated that all the examined variables had a significant impact on the EE%, as illustrated in Figure 1A–D.

The elevated EE% observed in the prepared PEG-CERs may be attributed to the incorporation of ceramide [16]. Owing to its high lipophilicity and structural compatibility with the lipid bilayer, ceramide can enhance membrane organization and lipid packing, thereby facilitating the accommodation and retention of the lipophilic drug within the bilayer [51,52] and providing a matrix suitable for drug encapsulation [52,53]. Furthermore, ceramides have been reported to increase membrane viscosity by forming gel-like domains and enhancing bilayer microviscosity [51], which is associated with their high phase-transition temperature [54]. Additionally, the pronounced lipophilicity of NIF (Log *p* = 4.43) favors its spontaneous integration into the lipid bilayer [24,38].

#### 3.2.1. Effect of Ceramide Type (X1)

In this study, three ceramides (III, IIIB, and VI) were used, each containing a sphingoid base, phytosphingosine, with an 18-carbon amino alcohol linked to a long hydrocarbon chain through an amide group. The phytosphingosine ceramides lack a 4,5 trans double bond and possess an extra hydroxyl group at the base chain 4 position. Therefore, they can be regarded as hydrated sphingosines [55]. Regarding ceramide type (X1), EE% was significantly enhanced (*p* = 0.0024) when ceramide VI was used compared with other ceramide types, following the order: ceramide VI > ceramide III > ceramide IIIB, as shown in Figure 1A.

Ceramide VI (α-hydroxy-N-stearoyl-phytosphingosine) is characterized by a saturated phytosphingosine backbone acylated with long-chain α-hydroxy stearic acid [16,56]. In contrast, ceramide III (N-stearoyl-phytosphingosine) consists of a phytosphingosine base linked to stearic acid [57] differ only by the presence of an additional hydroxyl group in ceramide VI. Meanwhile, ceramide IIIB (N-oleoyl-phytosphingosine) consists of a phytosphingosine base linked to oleic acid [57]. The molecular structure of ceramide III differs from that of ceramide IIIB in that the phytosphingosine backbone of ceramide IIIB is acylated with an oleoyl fatty acid, while ceramide III is acylated with a stearoyl fatty acid. Consequently, ceramide IIIB contains a cis double bond, while ceramide III, despite having the same number of carbon atoms, lacks a double bond [57].

Favorable ceramide-phosphatidylcholine dipolar matching in the liquid state might be one of the local criteria for tight molecular interactions. In contrast, unfavorable matching could clarify lateral domain segregation in ceramide-enriched gel phases [58]. The main phase transition temperature of hydrated ceramide III is observed at 110 °C, and ceramide IIIB at 88 °C [59], while ceramide VI is at 92.5 °C [57]. Since these long-chain ceramides (C18-ceramides) demonstrate notably high main phase transition temperatures, their presence is likely to cause considerable changes in the biophysical characteristics of phospholipid membranes [54]. Consequently, vesicle stability is enhanced, resulting in improved entrapment efficiency of the lipophilic NIF molecules.

Similar findings were observed from Takahashi and colleagues [54] demonstrated that a minor concentration (5%) of C18-ceramide elevated the main phase transition of lipids (1-palmitoyl- 2-oleoyl-sn-glycero-3-phosphoethanolamine (POPE) and 1-palmitoyl-2-oleoyl-sn-glycero-3-phosphocholine (POPC) from approximately 25 °C to physiological temperature (35–40 °C).

Also, Massey [60] reported that the incorporation of ceramide decreases the repulsion between the bulky headgroup, facilitating closer packing of the acyl chains. Additionally, he reported that ceramide elevates the transition temperature of phospholipids and hence increases the amount of gel phase phospholipid [60]. The high phase transition temperature facilitates the creation of less leaky and less permeable vesicles [61].

The balance between inter- and intramolecular hydrocarbon chain interactions, together with head group interactions, governs the order–disorder transition in ceramide systems [57]. Although ceramide VI and ceramide III molecules differ only by the presence of an additional hydroxyl group in ceramide VI, while their hydrophobic moieties remain similar, they exhibit markedly different physicochemical properties in terms of hydrogen bonding capacity and miscibility with other lipids [57,62,63]. The presence of the α-hydroxy fatty acid moiety in ceramide VI enhances its lipid miscibility by promoting stronger hydrogen bonding with adjacent lipids [64,65]. This improved miscibility between ceramides and phosphatidylcholine in lipid bilayers could enhance drug entrapment efficiency, particularly for hydrophobic compounds, by optimizing lipid packing and structural stability of the carrier system [66,67].

Rerek et al. [55] reported that phytosphingosine ceramides containing α-hydroxy fatty acid chains, such as ceramide VI, exhibit a weaker amide hydrogen bonding (intramolecular bonding) compared to their analogous ceramides lacking the α-hydroxy group, such as ceramide III and ceramide IIIB. Additionally, the incorporation of an additional α-hydroxy group into the ceramide fatty acid chain facilitates the exchange of both hydroxyl and amide hydrogen (intermolecular bonding) at reduced temperatures [55]. In contrast, ceramide III did not undergo either amide or hydroxyl hydrogen exchange except at very high temperatures. Furthermore, the incorporation of a cis double bond into the fatty acid chain of ceramide IIIB did not induce hydrogen exchange at significantly lower temperatures [55]. These findings indicate greater miscibility of ceramide VI with other lipids compared to ceramide III and ceramide IIIB, resulting in a significantly higher EE% of the lipophilic drug NIF.

A study conducted by Čuříková-Kindlová and colleagues [56] demonstrated that systems containing ceramide VI showed enhanced lipid miscibility compared to those containing ceramide III. This also agreed with another study in which Zbytovsk’ et al. [68] indicated a higher miscibility of ceramide VI with dimyristoylphosphatidylcholine (DMPC) compared to ceramide III, which was attributed to the higher hydrophilicity of the polar head group in ceramide VI relative to ceramide III, allowing the formation of more hydrogen bonds with the phospholipid headgroups compared to ceramide III, a property that is significant for the high miscibility of ceramide VI with phospholipids [68], as previously discussed.

In contrast, the limited miscibility of Ceramide III with other lipids could be attributed to its strong intramolecular hydrogen bonding capability [62], compared to the lower intramolecular bonding of ceramide VI. These properties might explain the tendency of ceramide III to crystallize and exhibit limited miscibility with other lipids [62]. Accordingly, Vovesn’ et al. [62] suggested that a substantial portion of ceramide III may not participate in vesicle formation due to its crystallization tendency, which could account for its lower entrapment efficiency compared to ceramide VI.

Therefore, differences in lipid miscibility may explain why ceramide VI generally provides superior lipid miscibility and, consequently, higher drug entrapment efficiency in PEGylated cerosomal formulations compared to ceramide III. Regarding ceramide IIIB, the existence of a double bond in its structure decreases the gel stability of the lipids [57]. Furthermore, the double bond in ceramide IIIB increases its hydration, allowing greater water penetration and resulting in lower EE% compared to ceramide III, which has a reduced level of hydration [55,69].

Ceramide III, IIIB, and VI, are known to have high critical packing parameters (CPP), typically around 1.2. This value is considerably higher than that of phosphatidylcholine (~0.7), allowing ceramide-rich vesicles to transition from spherical to flatter, more rigid, or tubular shapes [70]. Owing to the extra hydroxyl group, ceramide VI exhibits a higher packing parameter that promotes enhanced hydrogen bonding and tighter molecular packing, resulting in more stable vesicles. In contrast, ceramide III tends to form solid crystalline structures, making it less favorable for certain vesicular packing arrangements than ceramide VI [55,62].

Meanwhile, the presence of a double bond in ceramide IIIB increases its solubility but usually decreases packing efficiency compared to the saturated chains of ceramide III and ceramide VI [55,62]. Additionally, the presence of the unsaturated double bond in the carbon chain of ceramide IIIB induces a twisting effect in the molecule, resulting in less tightly packed cerosomes. This relaxed molecular packing produces a looser vesicle bilayer, which increases membrane permeability and facilitates drug leakage [16,28,41].

#### 3.2.2. Effect of Ceramide Amount (X2)

Increasing ceramide amount (X2) significantly enhanced the EE% of NIF (*p* = 0.0043), Figure 1B. This finding indicates that increasing ceramide content may improve the stability of the vesicle bilayer, thereby facilitating greater NIF encapsulation [71]. The direct connection between ceramide amount and the EE% may be attributed to the enhanced viscosity and lipophilicity of the formulation, which accompanies the higher ceramide content and subsequently hinders the diffusion of NIF, thereby ensuring its efficient entrapment within the vesicles [28,61,72]. It has been reported that increasing the ceramide amount elevates the phase transition temperature of phospholipids and enhances the ordering of the lipid bilayer, resulting in stronger intermolecular interactions and thus increasing EE%. This was in accordance with Massey [60] who reported that the increase in transition temperature of phospholipids was linear with the amount of ceramide in the bilayer. This also agrees with a study conducted by Takahashi et al. [54], where they found that the inclusion of ceramides (10, 20, 30 mol%) raised the lamellar gel-to-lamellar liquid crystalline phase transition temperature of POPC and POPE.

#### 3.2.3. Effect of Brij Type (X3)

Figure 1C shows that Brij type (X3) has a significant impact on EE% (*p*= 0.0014). The results indicate that PEG-CERs containing Brij 93 exhibited a significantly higher EE% (*p <* 0.05) compared with those containing Brij 35, which can be attributed to the difference in hydrophilic–lipophilic balance (HLB) values of the two edge activators. Surfactants with lower HLB values exhibit greater lipophilicity, making them more suitable for the incorporation of hydrophobic drugs [28,73], such as NIF. The HLB values of Brij 93 and Brij 35 are; 4 and 16.9, respectively [61,74].

A lower HLB value of Brij 93 corresponds to a longer alkyl chain, C_18_H_35_(OCH_2_CH_2_)_2_OH, which enhances the lipophilicity of the surfactant due to the formation of fewer hydrophilic voids within the bilayer, thereby reducing its fluidity [61] and inhibiting drug leakage, resulting in higher EE% values. Whereas Brij 35, consists of 23 units of polyethyleneglycol lauryl ether with the general formula of: C_12_H_26_ (CH_2_CH_2_O)_23_OH, and a high HLB value of 6.9 [74], indicating a lower proportion of hydrocarbon chains relative to the hydrophilic surface area [75].

Moreover, Brij 93 exhibits a greater critical packing parameter compared with Brij 35. The shorter polyoxyethylene chain of Brij 93 (2 units) compared with Brij 35 (23 units) [61,76] results in a smaller molecular volume, which contributes to a higher density at a given concentration and subsequently, a higher critical packing fraction [77].

#### 3.2.4. Effect of Sonication Time (X4)

Sonication time (X4) has also a significant influence on EE% (*p* < 0.0001), Figure 1D. While prolonged sonication without temperature control can lead to local heating and thermal fluidization of lipid bilayers [78], this effect was strictly mitigated in our study by performing the process in an ice bath to maintain a relatively low temperature. Furthermore, while the L-α-phosphatidylcholine component is intrinsically fluid (phase transition temperature of −10 °C) [79], formulations also incorporate different types of ceramides, which exhibit high phase transition temperatures [57,59]. Therefore, lipids with high phase transition temperature, as ceramides, confers significant rigidity and structural stability to the bilayer [80], making it highly resistant to thermal degradation under our cooled experimental conditions. Therefore, the observed reduction in EE% is attributed to extended sonication triggering the opening and shutting of the vesicles during the reformulation process [77], facilitating drug leakage with each opening. Moreover, it could be related to vesicle destruction, resulting in drug leakage [77]. The decreased EE% following sonication might be resulting from the formation of smaller vesicles entrapping lesser amount of drug [77,81].

### 3.3. Effect of Formulation Variables on VS (Y2)

The small particle size of the nano dispersion is crucial for creating a kinetically stable system that prevents particle aggregation and sedimentation. Moreover, the particle size of the nano-system could influence drug retention and the extent of skin permeation [28]. Table 2 illustrates that all the prepared formulae exhibited VS in the nano-range varying from 79.02 ± 0.58 to 372.4 ± 0.14 nm. The statistical analysis of VS data using ANOVA indicated that all the studied factors, except ceramide type (X1) (*p =* 0.3687), significantly influence the VS, (*p* < 0.05), as illustrated in Figure 2A–D.

#### 3.3.1. Effect of Ceramide Type (X1)

Regarding ceramide type (X1), no statistically significant effect on VS was observed (*p* = 0.3687), as shown in Figure 2A. This might be attributed to the minor differences in the molecular weights of the ceramides (ceramide VI, ceramide III, and ceramide IIIB, with molecular weights of 600 g/mol, 584 g/mol, and 582 g/mol, respectively [16,41]. Such small variations are unlikely to markedly affect the viscosity of the dispersion, which might explain the similarity in their VS values.

#### 3.3.2. Effect of Ceramide Amount (X2)

**Figure 5:**
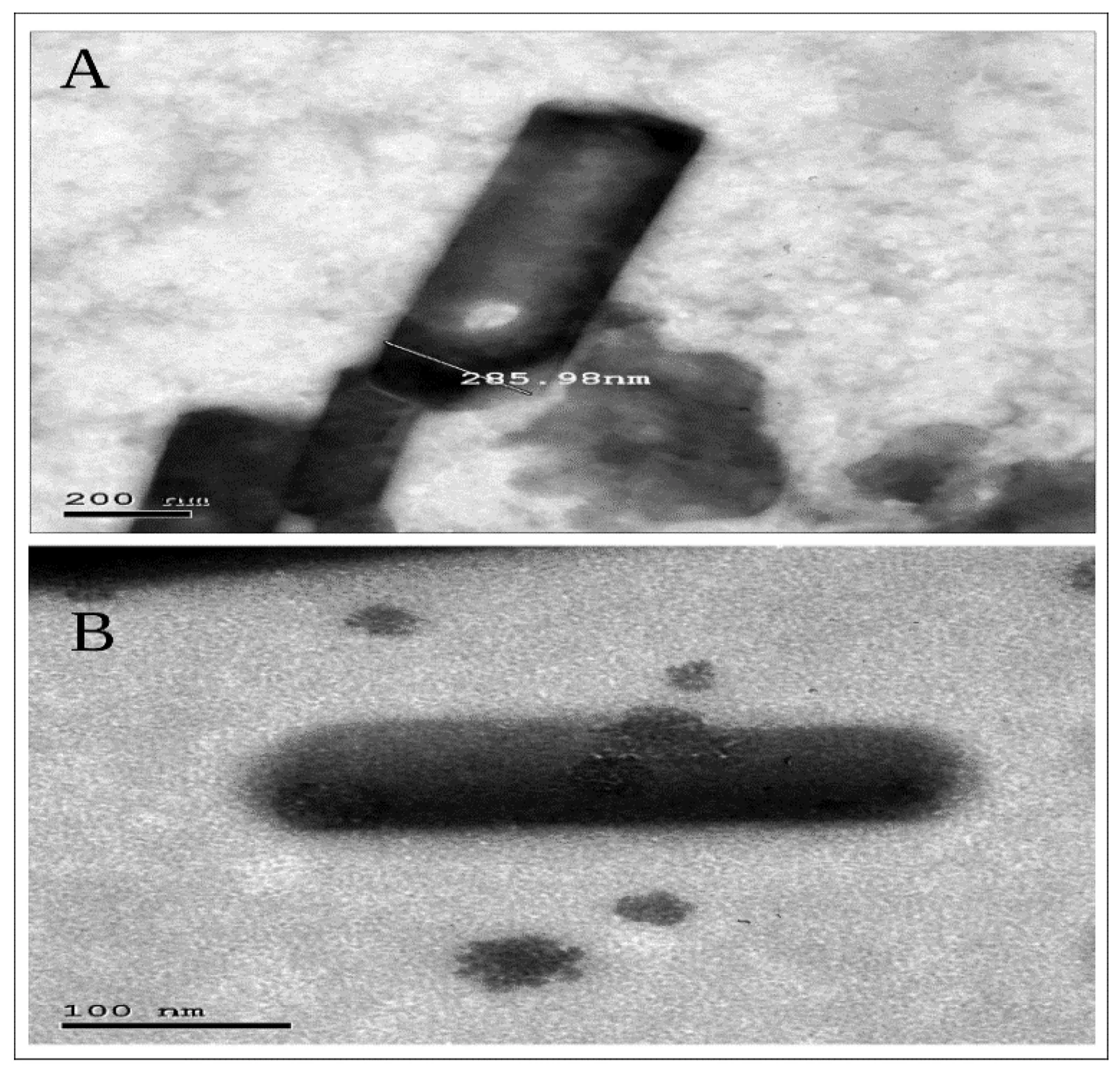
(**A**) TEM micrograph of the optimum C5 gel and (**B**) TEM micrograph of C6 subjected to ultrasonication for 10 min.

The VS of the formulated PEG-CERs significantly increased as the ceramide amount increased (*p* = 0.0316), Figure 2B. This effect might be attributed to the enhanced bilayer rigidity associated with increase in ceramide amount leading to stiffer and larger vesicles [71]. The direct connection between ceramide amount and VS could be explained based on two main factors. First, ceramides tend to aggregate in formulations [61,72] due to their small polar head group and substantial hydrophobicity [51], thereby increase in ceramide content could facilitate vesicle fusion or clustering, resulting in an overall increase in VS [71]. Second, ceramide-induced alterations in vesicle membranes which by turn contribute to VS enlargement. Ceramides exhibit a limited capacity to traverse the membrane leaflets, leading to their accumulation within the bilayer [82] promoting changes in membrane curvature, resulting in larger vesicle size [28]. This fact was confirmed via TEM later on (Figure 5A,B).

#### 3.3.3. Effect of Brij Type (X3)

Brij type (X3) significantly influenced the VS (*p* = 0.0080) (Figure 2C). PEG-CERs containing Brij 93 resulted in significantly larger VS compared to PEG-CERs containing Brij 35 which could be correlated with the PEG content of the two edge activators. Brij 93 contains 2 PEG units, while Brij 35 contains 23 PEG units [61,76]. The higher PEG content in Brij 35 might increase steric hindrance between vesicles, resulting in reduction in agglomeration, and consequently lower VS [83]. Additionally, increased hydrophilicity associated with higher PEG content might reduce the entrapment of the hydrophobic drug within CERs, contributing to a significant decrease in VS. In addition, a reduction in the PEG content of the PEGylated edge activator might enhance the rate of vesicle precipitation and agglomeration, leading to an increase in VS [73]. Additionally, the vesicle diameter is known to depend on the length of the alkyl chain of surfactants. As we previously mentioned, Brij-93 (C18) has a longer carbon chain length compared to Brij-35 (C12) [24,84]. Surfactants possessing longer alkyl chains typically result in larger vesicle formation [75]. These findings are consistent with the entrapment efficiency (EE%) results, indicating that PEG-CERs with higher EE% also exhibited larger VS. Surfactants with reduced PEG content exhibit greater lipophilicity (lower HLB value), promoting enhanced NIF entrapment and a subsequent increase in VS [61].

Furthermore, the pronounced difference in HLB between Brij 35 (~16.9) and Brij 93 (~4.0) can substantially influence their partitioning, interfacial organization, and supramolecular self-assembly within the lipid system. Brij 35 is highly hydrophilic and has a reported CMC of approximately 0.06–0.09 mM, favoring micellar or mixed-micellar organization [85,86,87]. In contrast, the lower HLB of Brij 93 indicates greater affinity for the hydrophobic lipid domain, favoring stronger membrane incorporation and modification of lipid packing and curvature [61,88].

This behavior is consistent with the inverse relationship between hydrophile–lipophile balance (HLB) and critical micelle concentration (CMC) established for the Brij homologous series, whereby a longer hydrocarbon chain and lower polyoxyethylene content markedly lower the CMC [89,90]. As Brij 93 combines a longer alkyl chain (C18) with a substantially lower HLB than Brij 35, it is expected to exhibit a considerably lower CMC and, therefore, a stronger thermodynamic tendency to remain incorporated within the assembling lipid bilayer rather than to partition into free aqueous micelles. This favors a shift in the supramolecular equilibrium of the system from small, highly curved mixed micelles, characteristic of the high-HLB, high-CMC Brij 35, toward larger, membrane-associated, tubulated cerosome structures for the low-HLB, low-CMC Brij 93, in agreement with comparable HLB-driven micelle-to-vesicle transitions reported for other Brij- and nonionic-surfactant-based vesicular systems [74,85,90,91,92,93]. Accordingly, this HLB/CMC-governed phase-level reorganization, rather than steric hindrance alone, represents the principal thermodynamic driver underlying the significantly larger VS obtained with the Brij 93-containing formulations.

Since the amount of the surfactants was fixed (each 5 mg) in the respective formulations, the observed differences in VS are not attributable to differences in surfactant concentration but rather to their distinct HLB-dependent interfacial and self-assembly behavior. Thus, Brij 35 may favor more highly curved and/or mixed-micellar structures, whereas Brij 93 may promote more membrane-associated and organized assemblies. The larger and more elongated structures observed for the Brij 93 formulation by TEM (Figure 5) are consistent with this difference in supramolecular organization. Therefore, the observed VS differences are likely governed by the combined effects of HLB-dependent surfactant partitioning, self-assembly, interfacial packing, and membrane curvature, along with steric hindrance [86,87,94].

#### 3.3.4. Effect of Sonication Time (X4)

Sonication of NIF-loaded PEG-CERs for 10 min resulted in a notable decrease in the VS (*p* < 0.0001), Figure 2D. This effect might be attributed to the exposure of vesicles to ultrasonic radiation, which promotes their dispersion into smaller sizes. The reduction in vesicle size induced by ultrasonic waves is also associated with enhanced vesicle deformability [81]. This process is governed by the generation and oscillation of cavitation bubbles in the liquid, induced by ultrasonic mechanical waves. At resonant frequencies, these bubbles undergo nonlinear oscillations followed by collapse. This collapse produces localized high temperatures, shock waves, and elevated pressures. Consequently, larger vesicles are disrupted in a random yet uniform manner by high-energy ultrasonic waves into smaller discoid fragments, which subsequently reorganize into thermodynamically stable vesicles [77,95,96].

### 3.4. Effect of Formulation Variables on PDI (Y3)

The PDI of the prepared NIF-loaded PEG-CERs ranged from 0.23 ± 0.0 to 0.49 ± 0.01 (Table 2), indicating a relatively homogeneous vesicle population, aligning with existing literature [71]. The high PDI values may be related to the irregular tubulated vesicular morphology of cerosomes [28,82] as confirmed later by TEM micrographs (Figure 5A,B).

ANOVA statistical analysis indicated that ceramide type (X1) and ceramide amount (X2) had a non-significant impact on PDI, Figure 3A,B, with *p* = 0.084 for ceramide type (X1) and *p* = 0.12 for ceramide amount (X2). In contrast, Brij type (X3) and sonication time (X4) had a notable impact on PDI, as shown in Figure 3C,D, with *p* < 0.0001 for Brij type (X3) and *p* < 0.0001 for sonication time (X4).

The use of Brij 93 was associated with higher PDI values in PEG-CERs, since the use of Brij 93 increased VS, as mentioned earlier, leading to a simultaneous increase in PDI values. This observation aligns with previous studies reporting that an increase in particle size in nano-dispersions is often associated with higher PDI values [41]. In this study Albash et al. [41] showed that the use of ceramide VI and a high phospholipid amount increased particle size, which results in a simultaneous rise in PDI values. Furthermore, this effect might also be attributed to the longer alkyl chain of Brij 93 (C18) [24] compared to Brij 35 (C12) [84], as longer carbon chains tend to increase the size distribution, resulting in higher PDI [24].

Regarding the effect of sonication time (X4), a reduction in VS was accompanied by a decrease in PDI, indicating enhanced homogeneity of the dispersion upon sonication. Similar findings were reported by Owodeha-Ashaka et al. [77], who applied 30 min of bath sonication to evaluate its effect on the stability-indicating characteristics of optimized pilocarpine hydrochloride-loaded niosomes for ocular drug delivery.

### 3.5. Effect of Formulation Variables on ZP (Y4)

Zeta potential measurement identifies the total surface charge of the formulated nano-dispersion, which is essential for determining its physical stability and anticipating potential interactions within the body [28]. An optimal surface charge enhances physical stability by generating electrostatic repulsion between vesicles, thereby preventing aggregation or fusion. All the fabricated PEG-CERs exhibited negative ZP values ranging from −21.4 ± 0.85 to −39.3 ± 0.28 mV (Table 2). These relatively high absolute ZP values indicate increased surface charge, reduced particle-particle interaction, and improved physical stability of the developed vesicles [82]. Statistical analysis using ANOVA test indicated that all the inspected factors, except the amount of ceramide (X2), had a significant influence on ZP values, as illustrated in Figure 4A–D.

#### 3.5.1. Effect Ceramide Type (X1)

Regarding Ceramide type (X1) (*p* = 0.0105), Figure 4A, NIF-loaded PEG-CERs formulated with ceramide VI exhibited significantly higher ZP values than those formulated with ceramide III and IIIB. In comparison to ceramide III and ceramide IIIB, ceramide VI tends to be more effective at increasing the negative surface charge of cerosomes. This is primarily attributable to the improved capacity of ceramide VI to participate in intermolecular hydrogen bonding within the bilayer, resulting in a more rigid and ordered structure that more effectively retains other negatively charged constituents compared to ceramide III and IIIB [55]. In addition, the incorporation of ceramide, particularly in high-binding formats (ceramide VI), alters the bilayer curvature from spherical to an elongated, tubulated form. This transformation is associated with the high packing parameter of ceramides, as previously discussed, which further reduces surface curvature and contributes to an increase in surface charge density [41,51,72].

Also, this might be ascribed to the amphiphilic nature of ceramide IIIB, which can adsorb onto the vesicle surface and form a shielding layer that masks the surface charge, thereby reducing zeta potential [28,82]. Moreover, the absolute ZP value of PEG-CERs containing ceramide IIIB was significantly higher than that of those containing ceramide III. This might be attributed to the presence of unsaturated fatty acids in ceramide IIIB, which enhance intermolecular repulsion between vesicles, thereby increasing the magnitude of the ZP [97].

#### 3.5.2. Effect of Ceramide Amount (X2)

Statistical analysis of the ceramide amount (X2) demonstrated a non-significant effect on ZP values (*p* = 0.5080), Figure 4B.

#### 3.5.3. Effect of Brij Type (X3)

Considering Brij type (X3), NIF-loaded PEG-CERs formulated with Brij 35 exhibited significantly lower ZP values than those formulated with Brij 93 (*p* < 0.0017), Figure 4C. Surfactants with higher PEG content tend to adsorb onto the surface of the nano-system, thereby reducing the apparent surface charge due to steric shielding effects [61]. The greater hydrophilicity of Brij 35 (HLB = 16.9; 23 PEG units) [74,84], in comparison with Brij 93 (HLB = 4; 2 PEG units), promotes its adsorption onto the vesicle bilayer, effectively masking the negative charge and resulting in lower ZP values [24].

Beyond simple steric masking by the PEG chains, the pronounced HLB divergence between Brij 35 and Brij 93 is expected to govern the electrokinetic behavior of the PEG-CERs through the same CMC-dependent partitioning and self-assembly differences discussed for VS (Section 3.3.3). The high-HLB, high-CMC Brij 35 favors a more hydrophilic, expanded, loosely associated PEG corona at the vesicle-water interface, consistent with mixed-micellar-like organization [89,91,92]. Such an extended, hydrated, ion-permeable corona displaces the electrokinetic shear (slipping) plane further from the charged bilayer surface and screens the underlying negatively charged phosphate and ceramide head-groups, both of which act to lower the measured ZP of PEGylated colloidal carriers [91,98]. In contrast, the low-HLB, low-CMC Brij 93 is retained more deeply within the hydrophobic bilayer core, forming a thinner, more compact interfacial layer that leaves the native surface charge comparatively less shielded and keeps the shear plane closer to the vesicle surface, thereby preserving a higher absolute ZP. This CMC- and HLB-dependent difference in interfacial organization, consistent with reports for other Brij-based nonionic-surfactant vesicular systems of varying HLB [91,92,93], therefore complements, rather than replaces, the steric-shielding explanation and provides a more complete, phase-behavior-based rationale for the significantly lower ZP observed with Brij 35 relative to Brij 93.

#### 3.5.4. Effect of Sonication Time (X4)

Statistical data, Figure 4D, indicated a substantial reduction in the ZP values of the formulated PEG-CERs after 10 min of ultrasonication (*p* < 0.0001), suggesting that sonication-induced changes in VS and structure may be accompanied by alterations in the surface characteristics of the vesicles, suggesting a potential relationship between variations in VS and ZP. The reduction in zeta potential following ultrasonication may be associated with physical fragmentation and reorganization of the PEG-CERs vesicular structure, consistent with the morphological changes [99] observed in the TEM images (Figure 5B). In addition, sonication-induced redistribution or partial desorption of the PEGylated surfactant at the vesicle surface may contribute to changes in the interfacial characteristics and the measured ZP. These phenomena are primarily driven by the mechanical stresses of acoustic cavitation, which can induce membrane deformation, permeabilization, and vesicular disruption [100,101,102]. Consistently, PEGylated vesicular systems have been reported to exhibit heightened susceptibility to ultrasound-induced membrane permeabilization and structural disruption [100,103].

This was similarly noted in a previous study conducted by Floris et al. [104], who examined the impact of various ultrasonic radiation modalities on the mean diameter and PDI of chitosan nanoparticles synthesized using the ionotropic gelation technique. Likewise, Hussain et al. [99] optimized the synthesis conditions of chitosan tripolyphosphate (TPP) nanoparticles, including ultrasonication (0–12 min), to achieve minimum particle size, optimum zeta potential, along with a narrow PDI. Both studies demonstrated that ultrasonication can induce significant polymer degradation, primarily due to cavitation effects.

Finally, ultrasonication can induce substantial polymer degradation, primarily due to cavitation effects. Moderate ultrasonication reduces particle size by disrupting aggregates, whereas prolonged ultrasonication may cause extensive fragmentation of the compact nanoparticle structure [99]. These findings were further supported by TEM micrographs of formulations C5 and C6 (Figure 5A and B, respectively).

### 3.6. Optimization of the Prepared NIF-Loaded PEG-CERs

The optimal levels of the independent variables were determined by analyzing the dependent responses utilizing Design Expert^®^ software. Analysis results selected C5 as the optimal formula, achieving the highest desirability value (0.795) and satisfying the predefined criteria of maximum EE%, minimum VS, with PDI and ZP maintained within acceptable ranges. The selected optimum formula was further developed utilizing 10mg of ceramide VI and 5mg of Brij 93, without sonication. The optimized prepared formula showed a VS of 292.95 ± 0.78 nm with a high NIF EE% of 96.71 ± 0.0% *w*/*w*, a ZP value of −37.5 ± 0.57 mV, and an acceptable PDI value of 0.47 ± 0.0. A strong correlation was observed between the observed and predicted outcomes of C5 (Table 4). Accordingly, the optimum NIF-loaded PEG-CERs formula (C5) was chosen for additional investigations.

### 3.7. Characterization of the Optimum Selected PEG-CERs Formula

#### 3.7.1. Transmission Electron Microscopy (TEM)

TEM is essential for studying the morphology of the developed system and for corroborating the findings obtained from vesicle size measurement via Malvern Zetasizer [9]. Formulas C5 and C6 were chosen for TEM examination to investigate the impact of sonication on PEG-CERs characteristics. As shown in Figure 5A,B, the PEG-CERs predominantly exhibited an elongated tubular morphology. This elongation might be explained by the fact that enriching phosphatidylcholine with ceramide resulted in the elongation of their vesicles, which is associated with the partitioning of ceramide within the phosphatidylcholine bilayer and the subsequent rigidification of the interface.

The elevated packing parameter of ceramide relative to that of phosphatidylcholine resulted in the flattening of the phosphatidylcholine bilayer curvature during vesicle preparation (confirming the miscibility between ceramide VI and phosphatidylcholine). The occasional existence of spherical vesicles alongside tubules may result from the non-uniform distribution of ceramide within the bilayer, leading to ceramide-rich domains exhibiting flat morphology and ceramide-poor domains having spherical morphology [41,51,72]. These observations are consistent with previous reports on ceramide VI-based CERs. Furthermore, Figure 5B illustrates that extensive ultrasonication induces significant fragmentation of the compact vesicle structure, resulting in marked reductions in both ZP and VS, as mentioned before, which was aligned with findings described by Floris et al. [104] and Hussain et al. [99].

#### 3.7.2. Differential Scanning Calorimetry (DSC)

**Figure 6:**
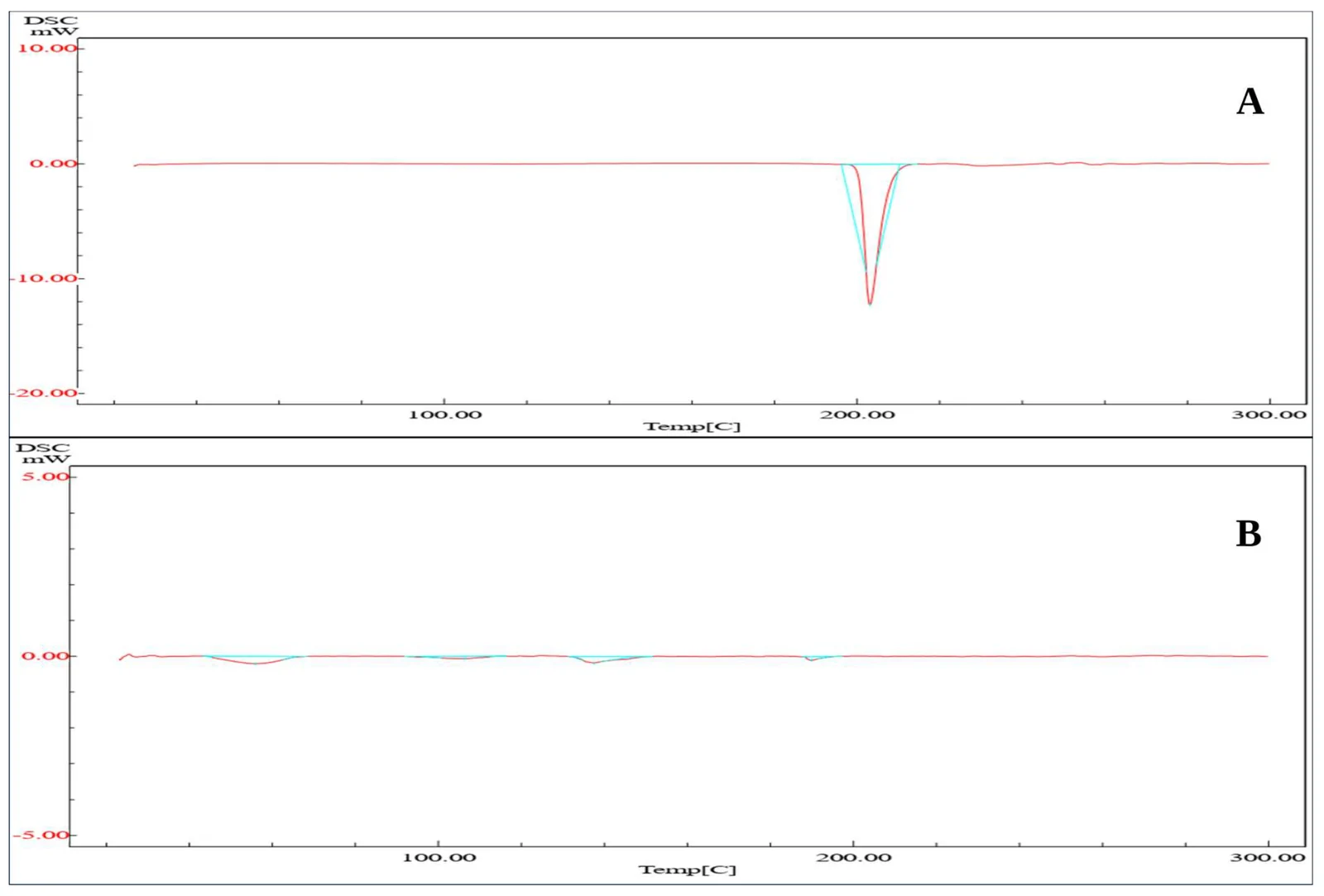
Differential scanning calorimetry (DSC) thermograms of A) pure NIF and B) lyophilized NIF-loaded PEG-CERs optimized formula (C5) separately.

Figure 6A presents the DSC thermograms of pure NIF and the lyophilized form of the optimal formula C5. DSC of pure NIF possessed a sharp endothermic peak at 203.18 °C, which corresponds to its melting point, which is consistent with previously reported values in the literature [105]. This sharp peak completely disappeared in the thermogram of the optimal formula C5, Figure 6B. The absence of the characteristic NIF endothermic peak in CERs indicates amorphization of the drug, which can enhance its solubilization and confirms successful encapsulation within the vesicles [38].

#### 3.7.3. Fourier-Transform Infrared Spectroscopy (FTIR)

**Figure 7:**
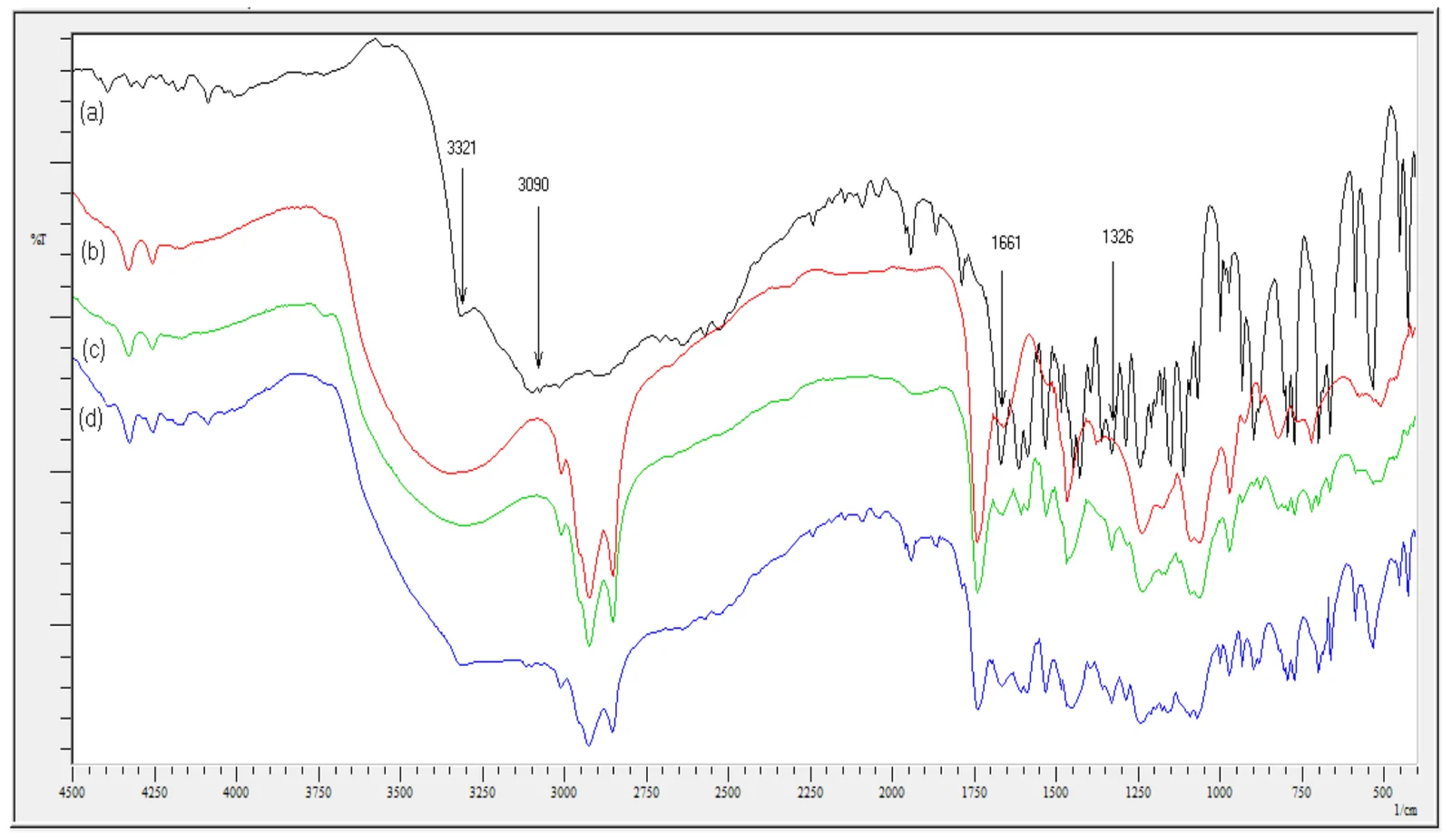
ATR-FTIR spectrum of: (a) pure NIF, (b) Blank formula C5, (c) lyophilized NIF-loaded PEG-CERs optimum formula C5, and (d) physical mixture of NIF and the used excipients.

Fourier-transform infrared (FTIR) spectroscopy was utilized to evaluate potential intermolecular interactions between NIF and the selected excipients in the optimized PEG-CERs formulation. Figure 7 demonstrates the FTIR spectrum for pure NIF, blank C5 formulation, a physical mixture of NIF with the C5 excipients, and the lyophilized optimum C5 formula. The NIF spectrum showed distinct bands corresponding to its chemical structure, particularly, an N–H stretching vibration at 3321 cm^−1^, a broad signal attributed to the aromatic C–H stretching at 3090 cm^−1^, a C=O stretching vibration at 1660 cm^−1^, and a CF3 group signal at 1326 cm^−1^ [23].

The FTIR spectrum of the optimum C5 formulation exhibited notable attenuation and the complete disappearance of various characteristic NIF peaks, indicating successful drug entrapment within the vesicular matrix. For the physical mixture of NIF and excipients, the characteristic NIF peaks appeared at their original positions without detectable shifts or new peak emergence; this confirms the absence of covalent chemical interactions and demonstrates the physicochemical compatibility of NIF with the formulation components. Additionally, the FTIR spectrum of the physical mixture exhibited peaks with less intensity when compared to pure NIF and the blank C5 formulation, which can be attributed to a dilution effect [106]. Collectively, these spectral findings substantiate the molecular compatibility among the constituents and verify stable vesicle formation.

### 3.8. Short-Term Physical Stability Study Tests

Stability studies demonstrated that the optimal C5 formula exhibited good storage stability throughout the studied storage period (3 months). Moreover, physicochemical evaluations revealed non-significant variations (*p* > 0.05) in EE%, VS, PDI, and ZP as well as no change in appearance, color, and no sign of agglomeration or aggregation of the stored vesicles compared to the freshly prepared samples, as demonstrated in Table 5. The observed stability might be related to the existence of Brij 93, which likely enhances vesicle integrity due to the ether bond in its chemical structure. Ether bonds are more resistant to hydrolysis in aqueous environments than the ester bonds found in other non-ionic surfactants, thereby reducing the risk of degradation and improving the stability of colloidal structures [34].

### 3.9. Ex Vivo Skin Permeation Study

**Figure 8:**
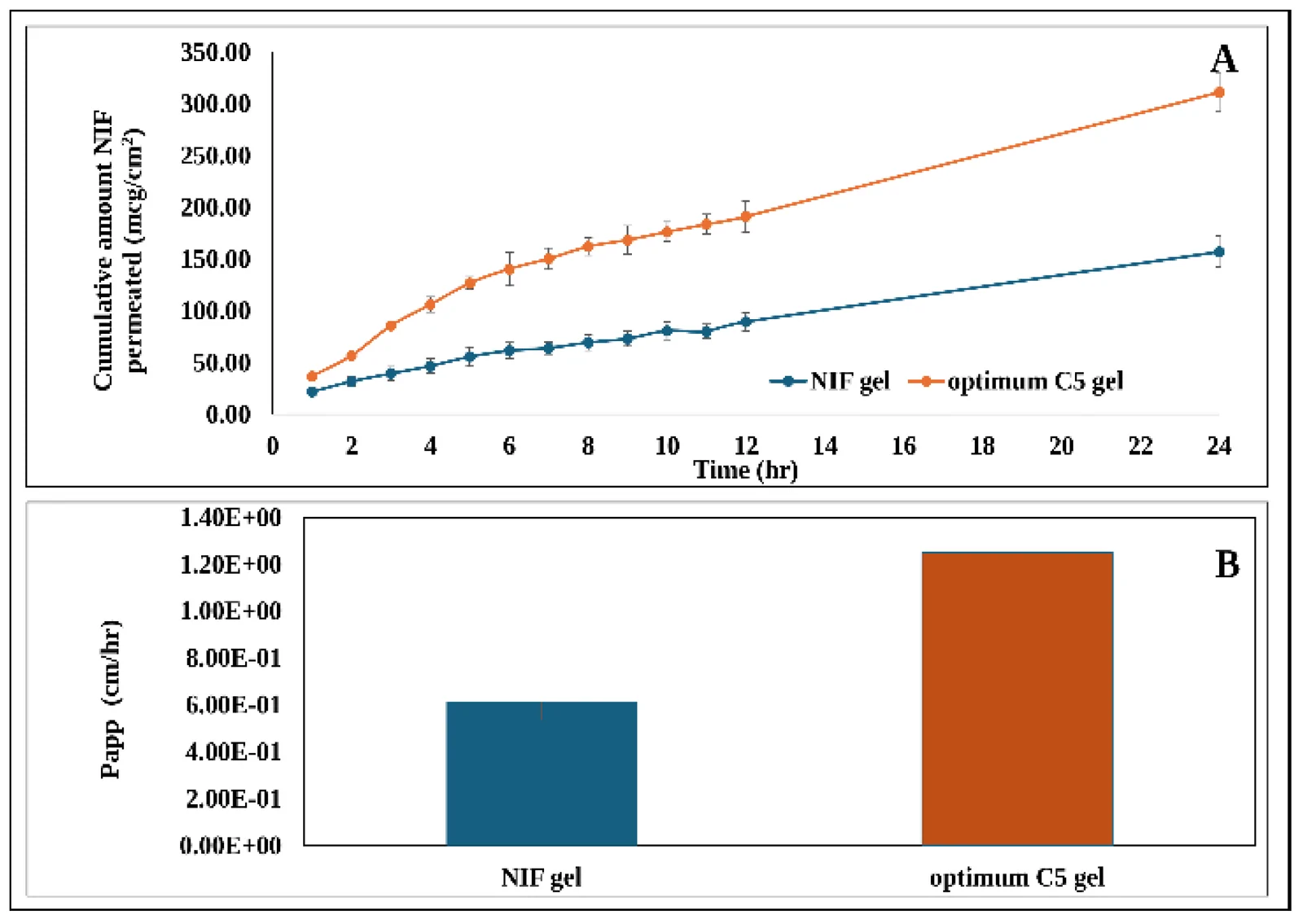
Ex vivo skin permeation profile of NIF through rat skin from the optimized C5 gel compared to NIF gel. (**A**) cumulative amount of NIF permeated (mcg/cm^2^) ± SD from the optimized C5 gel compared to NIF gel, and (**B**) permeation coefficient (*P*_app_ ± SD) from the optimized C5 gel compared to NIF gel.

A 24-h permeation study was conducted to compare NIF permeation from the optimal C5 formula and pure NIF gel formulations. Figure 8 presents the permeation flux, including both the cumulative amount permeated and the apparent permeability coefficient (*P*_app_) of NIF across rat skin. Figure 8A shows that the cumulative amount of NIF permeated from the C5 gel exhibited superior results compared to pure NIF gel along the whole permeation study time.

Figure 8B demonstrates that both the permeability coefficient (*P*_app_) and steady-state flux (J_ss_) of C5 optimum gel were significantly (*p* < 0.05) higher by 2.02-folds compared to pure NIF gel. Specifically, the *P*_app_ was 1.30 ± 0.075 cm/hr for the C5 gel, compared to 0.65 ± 0.063 cm/hr for NIF gel. Regarding J_ss_ results, values of 11.38 ± 0.634 µg/cm^2^.hr and 5.63 ± 0.417 µg/cm^2^.hr were calculated corresponding to C5 and NIF gel formulae, respectively indicating enhanced permeability of NIF from the optimal gel formula C5.

Niflumic acid (NIF) is being classified as a Biopharmaceutical Classification System (BCS) class II, indicating its low solubility in water. This suggests that the rate-limiting step in its transdermal delivery depends on its partitioning from the stratum corneum into the viable epidermis. Accordingly, vehicles with varying lipophilicities can enhance partitioning and increase flux across the stratum corneum [23,107].

The significant enhancement in NIF permeability from the prepared PEG-CERs (C5 gel formulation) through rat skin, compared to pure NIF gel formulation, might be attributed to the increased lipophilicity of CERs, facilitating their interactions with lipophilic cell membranes and promoting cellular uptake of C5 [82,108,109]. Furthermore, the structural similarity between synthetic ceramide VI and the natural skin lipid (ceramide 7) enhances penetration through the stratum corneum by disrupting intercellular lipid organization, thereby facilitating the transport of active drugs [51].

Siskind and Colombini [110] reported that ceramide-induced permeabilization, both C2- and C16-ceramide, occurs through the creation of large, stable ceramide channels, which are subsequently associated with apoptosis. Also, Contreras et al. [111] reported that long-chain ceramide’s geometry is favorable to the formation of inverted hexagonal phases in the membrane and promotes negative curvature. In addition, ceramides might increase membrane curvature and permeability via trans-bilayer lipid movement, a phenomenon known as flip-flop [30,112]. This phenomenon was ascribed to the propensity of ceramide to differentiate into domains, generate negative spontaneous curvature, and the enhanced bending rigidity of ceramide-enriched domains, resulting in bilayer invagination and vesiculation [113].

It has been indicated that ceramides incorporated into the phospholipid membrane can lead to transbilayer lipid movement, destabilize phospholipid membranes, leading to leakage, fusion, and budding of vesicles (fission) [68,110,113]. Recent research indicates that ceramide may alter the membrane’s physical state. Numerous studies have demonstrated that the creation of ceramide within the membrane results in the segregation of this lipid into Cer-rich domains, which form large platforms or rafts in which various cell surface receptors oligomerize [111]. These rigid domains are attributed to strong intermolecular interactions among ceramide molecules, resulting in a more compact membrane structure [111]. Another notable characteristic of ceramide is its ability to influence overall membrane curvature and stability, likely due to its intrinsic negative curvature, which promotes the creation of non-lamellar structures. Ceramide is therefore proposed to induce vesicle efflux, membrane fusion, and vesicle budding through the transient formation of non-bilayer intermediates [111].

Two main mechanisms have been proposed to explain ceramide-induced trans-bilayer lipid movements. The first is ceramide’s ability to promote the transition from lamellar to non-lamellar phases. The development of ceramide on one side of the membrane is proposed to trigger the transient formation of non-lamellar structural intermediates. This process might result in the loss of the asymmetry of the membrane bilayer, mixing of surrounding lipids, and, as the structure collapses, trans-bilayer lipid exchange. The second mechanism is related to the general physical properties of membranes, including mass conservation within each leaflet. The enrichment of one membrane layer with ceramides results in their diffusion to the other leaflet, which in turn prompts the movement of non-ceramide lipids in the opposite direction, thereby preventing net mass transfer between monolayers. Observations of ceramide flip-flop are particularly relevant to its biological effects in mammalian cells, notably within the sphingomyelinase (SMase) signaling pathway [113]. Additionally, the high elasticity of the prepared PEG-CERs might contribute to this effect, as the incorporation of Brij-93 enhances their ability to deform and penetrate the stratum corneum, thereby facilitating deeper delivery of the encapsulated drug into deeper skin layers [24].

Crucially, in the context of our study design, the primary downstream therapeutic target was solid Ehrlich carcinoma (SEC)—a tumor model established via subcutaneous inoculation beneath the epidermal and dermal layers. To exert effective local therapeutic activity against such deep-seated subcutaneous lesions, the topically applied formulation must achieve efficient full-thickness transcutaneous flux rather than remaining confined solely to the superficial stratum corneum. Therefore, the elevated J_ss_ and *P*_app_ values obtained here reflect the requisite transdermal transport capacity needed to access the underlying tumor bed. Nevertheless, detailed layer-specific skin biodistribution remains an informative direction for ongoing mechanistic investigation.

### 3.10. In Vivo Anticancer Efficacy of the Optimum C5 Gel

Ehrlich tumor cells are typically used as experimental tumors to study the antitumor effectiveness of different drugs. The rational utilization of Ehrlich tumor cells for in vivo investigations is supported by various pivotal reasons, including simplicity of implantation of a standard number of cells in different mice strains, ease of tumor growth quantification, and reduction in experimental duration time [114]. Furthermore, Ehrlich carcinoma is similar to human tumors’ vulnerability to chemotherapy, as it is an undifferentiated carcinoma characterized by a rapid growth rate [115]. Niflumic acid, alongside its anti-inflammatory properties, has exhibited an anti-proliferative effect in different tumor cell types [116].

#### 3.10.1. Effect on Survival Rate, Tumor Volume, and Tumor Inhibition Rate

In the present study, SEC-bearing mice treated with the optimum C5 gel (Group V) exhibited a significant increase in the survival rate and a marked decrease in the tumor volume (*p* < 0.05). The survival rate in the SEC control group (Group II) was 60%, 70% in both the NIF gel-treated group (Group III) and the blank C5 gel-treated group (Group IV), and 80% in the optimum C5 gel-treated group (Group V) compared to 100% survival in the negative control group (Group I).

**Figure 9:**
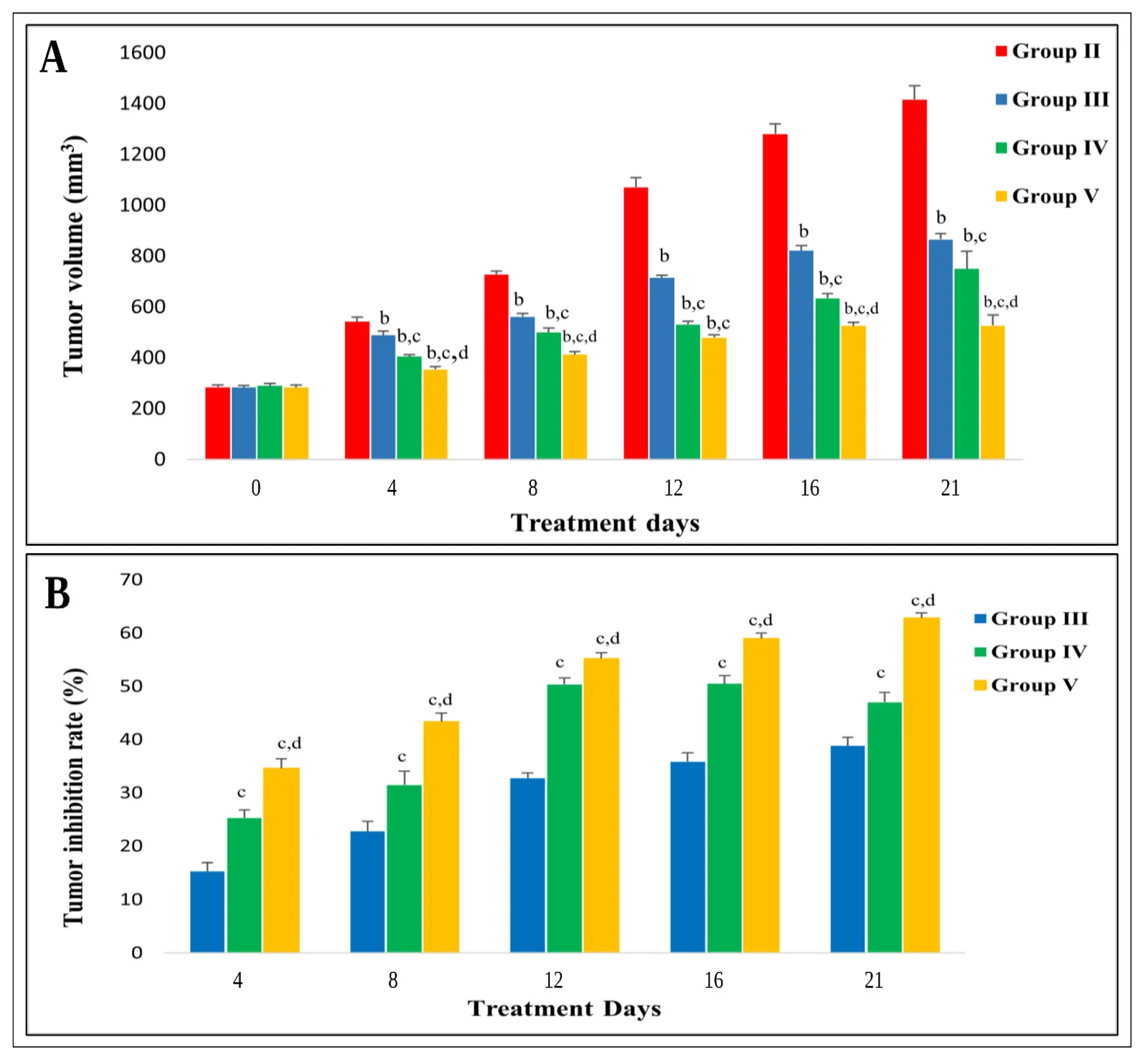
Effect of C5 gel treatment of SEC-bearing mice on: (**A**) tumor volume and (**B**) tumor inhibition rate. Data are expressed as mean ± SEM. The data were analyzed using one-way ANOVA followed by Tukey’s multiple comparisons test. A difference with *p* < 0.05 was considered to be statistically significant. ^b^ statistically significant from the SEC control (Group II). ^c^ statistically significant from SEC bearing mice receiving NIF gel (Group III), ^d^ statistically significant from SEC bearing mice receiving blank C5 gel (Group IV). SEC: Solid Ehrlich carcinoma, NIF: niflumic acid.

Assessment of tumor volume can be utilized to predict cancer progression [117]. In the current study, tumor volume was assessed throughout the treatment period, on days 1, 4, 7, 10, 15, and 21 (the day of termination), Figure 9A. Following tumor induction, tumor volumes increased in all groups. Tumor volume in the SEC control group was 284.2 ± 8.2 mm^3^ on the 1st day of treatment and increased gradually till reaching 1416 ± 54.6 mm^3^ on the 21st day of treatment, exhibiting the greatest tumor volume than all other treated groups. On the other hand, the optimum C5 gel-treated group exhibited a substantial decrease (*p* ˂ 0.05) in mean tumor volume, showing the least tumor volume of 526.8 ± 41.7 mm^3^ compared to the SEC control group 21 days post-treatment. The tumor progression rate was significantly slower in both the NIF gel-treated group and the blank C5 gel-treated group than the SEC control group, having tumor volumes reaching 865.9 ± 22.2 and 749.9 ± 68.6 mm^3^, respectively, on the 21st day post-treatment.

Regarding tumor inhibition rate (TIR), treatment with the optimum C5 gel exerted 62.8% inhibition of tumor growth versus 38.85% in the NIF gel-treated group and 47% in the blank C5 gel-treated group, respectively. Figure 9B. These outcomes were in line with several studies showing that NIF can inhibit the growth of nasopharyngeal, breast, and lung carcinoma cells successfully [18,20,21]. In addition, ceramide VI, a cell-permeable ceramide, has shown substantial anti-tumor activity against various cancer cell lines [118]. Consequently, our results prove the augmented antitumor activity and tumor growth inhibitory effect achieved by the optimum C5 gel treatment.

#### 3.10.2. Effect on Tumor EGFR, ERK 1, ERK 2, and miR-21-5p Gene Expression

Epidermal growth factor receptor (EGFR), a vital upstream molecule in the RAS/RAF/MEK/ERK pathways, plays a crucial role in the regulation of normal cell survival, proliferation, and differentiation [119]. Furthermore, the signal transduction pathway phospho-EGFR → ERK1/2 has been reported to have a crucial role in maintaining the normal epidermal cell proliferation [120]. Aberrant expression of EGFR and its subsequent activation of RAS/RAF/MEK/ERK cascade is a common mechanism for cancer cell survival, proliferation, invasion, angiogenesis, and metastasis [11].

**Figure 10:**
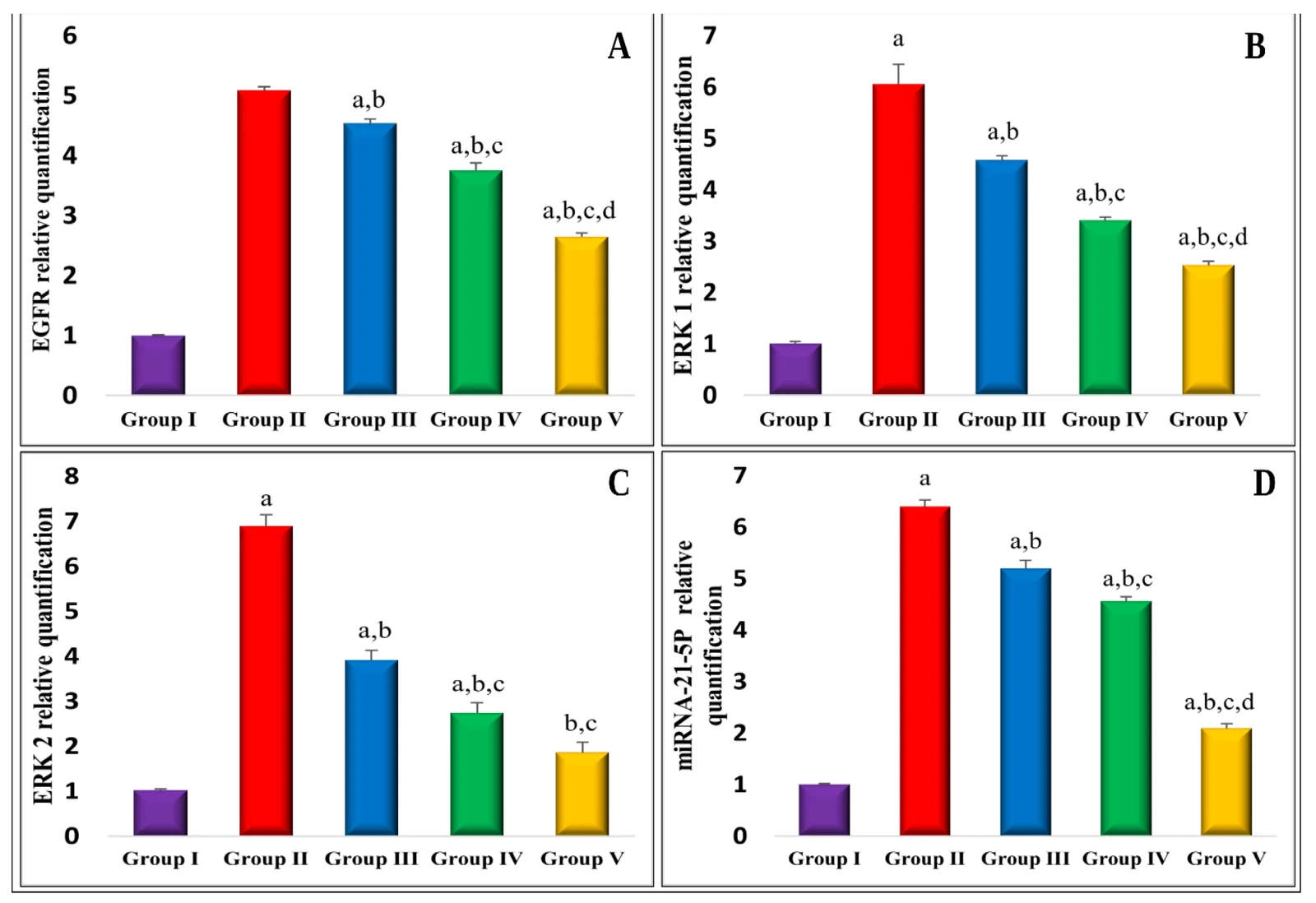
Effect of optimum C5 gel treatment of SEC bearing mice on: (**A**) EGFR, (**B**) ERK 1, (**C**) ERK 2, and (**D**) miRNA-21-5p gene expression. Gene expression levels of EGFR, ERK 1, ERK 2, and miRNA-21-5p are expressed as relative quantification mean ± SEM using GAPDH as reference gene and Group I (normal control) as the calibrator for relative fold change values. The data were analyzed using one-way ANOVA followed by Tukey’s multiple comparisons test. A difference with *p* < 0.05 was considered to be statistically significant. ^a^ statistically significant from normal control (Group I). ^b^ statistically significant from SEC control (Group II). ^c^ statistically significant from SEC bearing mice receiving NIF gel (Group III). ^d^ statistically significant from SEC bearing mice receiving blank C5 gel (Group IV). EGFR: epidermal growth factor receptor, ERK 1: extracellular signal-regulated kinase 1, ERK 2: extracellular signal-regulated kinase 2, SEC: Solid Ehrlich carcinoma, NIF: niflumic acid.

In the present study, gene expression of EGFR was significantly upregulated in the SEC control group by 5.1-fold in comparison to the normal control group (Figure 10A). This aligns with several studies reporting EGFR upregulation in several human malignancies and its correlation with poor prognosis for melanoma, head and neck squamous cell carcinoma, breast cancer, and non-small cell lung carcinoma (NSCLC) [121,122]. Conversely, treatment with both NIF gel and blank C5 gel led to a significant (*p* < 0.05) downregulation of EGFR by 10.9% and 26.4%, respectively, compared to SEC control animals (Figure 10A). Furthermore, the optimum C5 gel treatment produced the most profound impact, verified by a significant (*p* < 0.05) downregulation of EGFR by 47.9% in comparison to the SEC control group, which suggests NIF interference with EGFR signaling at a transcriptional level.

The inhibitory effect of NIF on EGFR expression might be mediated indirectly through its suppression of inflammatory signaling pathways, particularly COX-2-PGE_2_-dependent mechanisms and attenuation of ERK1/2 and nuclear factor kappa B (NF-κB)-dependent signaling. Although EGFR signaling is classically recognized as an upstream regulator of COX-2 expression [123]. Multiple lines of evidence indicate that COX-2–derived PGE_2_ could in turn, promote multiple tumor-supportive functions, including stimulation of tumor growth via upregulation of EGFR–ERK1/2 signaling cascade, thereby reinforcing the oncogenic drive of EGFR signaling and sustaining a tumor-promoting inflammatory microenvironment [123,124].

Similarly, Bazzanai et al., [125], reported that PGE_2_ promotes EGFR nuclear translocation, alters gene expression, and induces tumor cell progression in lung adenocarcinoma cells. Moreover, EGFR-associated signaling might be suppressed by ceramide-mediated pathways [126]. Although direct evidence for ceramide VI alone suppressing EGFR expression remains limited, it has been suggested that ceramide VI can promote EGFR/HER receptor internalization and degradation, thereby attenuating downstream proliferative pathways, including ERK activation [126].

The extracellular signal-regulated kinases ERK 1 and ERK 2, subfamilies of the MAPKs, are ubiquitous serine-threonine kinases that transduce signals participating in the regulation of various physiological processes, which include cell growth, survival, proliferation, adhesion, differentiation, and transcription [127]. Our results revealed a significant gene expression upregulation of both ERK 1 (Figure 10B) and ERK 2 (Figure 10C) by 6.1 and 6.8-fold, respectively, in the SEC control group compared to normal control animals. These results align with previous studies, which demonstrated increased ERK expression in a variety of human tumors, including breast, ovarian, lung, and colon cancer [11].

Consistently with EGFR downregulation, treatments with NIF gel, blank C5 gel, and the optimum C5 gel formulations substantially downregulated gene expression of both ERK 1 by 25.7%, 43.8%, and 58.2%, respectively (Figure 10B), as well as ERK 2 by 43.2%, 60.3%, and 73%, respectively (Figure 10C), compared to the SEC control group. Notably, the optimal C5 gel exhibited the most pronounced effect, supporting effective suppression of EGFR/ERK signaling cascade. These outcomes aligned with a prior study that illustrated the anti-tumor activity of NIF in nasopharyngeal carcinoma cells, where ERK1 was found to bind directly with NIF, resulting in down-regulation of ERK1/2 and suppression of downstream proliferative pathways [21].

MicroRNA-21 (miR-21), found at 17q23.1, is an oncogenic microRNA that modulates multiple tumor suppressor genes and proteins, including PTEN, PDCD4, TPM1, TIMP3, and RHOB by promoting their translational repression or degradation [128]. In addition, miR-21 plays a role in key signaling pathways, including Wnt/β-catenin, PTEN/PI3K/AKT, and RAS/MEK/ERK, contributing to tumor proliferation, survival, and invasion [129]. In the current work, miR-21-5p gene expression (Figure 10D) was markedly upregulated by 6.4-fold in SEC control animals compared to the normal control group. This aligns with several studies demonstrating miR-21-5p overexpression in numerous human cancers, including prostate, breast, and gastric cancer, highlighting its importance in tumor progression in NSCLC and ovarian cancer [130], being identified as the environmental driver of malignant melanoma [131]. Similarly, miR-21-5p has been noted to be overexpressed in cancerous melanocytic skin tissues, enhancing cell proliferation and the G1/S transition [132].

The observed upregulation of miRNA-21-5p expression might be attributed to transcriptional upregulation of EGFR and ERK1/2. Previous studies demonstrated that miRNA-21-5p is transcriptionally upregulated downstream of EGFR signaling via ERK1/2-dependent activation of transcription factors AP-1 and β-catenin [133,134]. Further evidence indicates that once upregulated, miRNA-21 could modulate EGFR /Akt signaling via targeting negative regulators such as Sprouty2 (Spry2), von Hippel–Lindau (VHL), and peroxisome proliferator-activated receptor alpha (PPAR-α).

In addition, miRNA-21-mediated downregulation of Spry2 reinforces Ras/ERK signaling and enhances malignancy in PDAC models, creating a positive feedback loop that enhances proliferation, survival, and invasiveness in tumor cells [134]. Therefore, our findings suggest that alterations in EGFR and ERK1 expression might influence miR-21-5p levels, providing a molecular link between upstream EGFR/ERK1/2 expression and downstream oncogenic microRNA regulation.

On the contrary, our results reveal that SEC-bearing animals’ treatments with NIF gel, blank C5 gel, and the optimum C5 gel resulted in a marked downregulation of miRNA 21-5p by 18.9%, 28.8%, and 67.3%, respectively (Figure 10D) compared to SEC control animals. The present study is the first one to demonstrate the impact of the optimum C5 gel on the EGFR/ERK pathway and the modulation of miR-21-5p as a possible mechanism for its anticancer effects.

#### 3.10.3. Effect on Tumor Cyclin D1, MMP-2, and COX-2 Levels

Cyclin D1, a crucial regulator of cell cycle progression, integrates extracellular mitotic signals into DNA synthesis through binding to cyclin-dependent kinase 4/6 (CDK4/6), thereby facilitating the transition of the cell cycle from G1 to S phase [135]. As shown in Table 6, cyclin D1 level was significantly increased by 4-fold in the SEC control group compared to the normal control group. These results comply with several lines of evidence reporting that high expression of cyclin D1 triggers unchecked cellular proliferation, thereby facilitating tumor growth [136]. As a result, cyclin D1 is considered an oncogenic driver in various types of cancers, such as lung cancer, breast cancer, melanoma, and cutaneous squamous cell carcinoma [135]. Furthermore, in melanoma, elevated Cyclin D1 levels have been linked to tumor progression, increased Breslow thickness, and resistance to targeted therapies, highlighting its role in tumor aggressiveness and therapeutic response [137].

The anti-proliferative, cell cycle-blocking effect of NIF, particularly in the optimal C5 gel, was associated with a significant decrease in cyclin D1 levels, as observed in the present study (Table 6). Upon treatment of SEC-bearing mice with NIF gel, blank C5 gel, and the optimum C5 gel, cyclin D1 level decreased by 26.7%, 43.4%, and 61.8%, respectively, compared to the SEC control group. This is in accordance with a previous study demonstrating NIF-mediated reduction in cyclin D1 expression in neuroblastoma, which was associated with cell cycle arrest in the G1 phase and induction of apoptosis [138].

Moreover, a former study on pancreatic cancer cells has shown that ceramide VI inhibits proliferation by reducing cyclin D1 expression levels with subsequent G1 phase cell cycle arrest [139]. NIF has been shown to suppress EGFR and ERK1/2 expressions, leading to the suppression of downstream proliferative mediators, including cyclin D1, which is transcriptionally regulated by ERK via multiple mechanisms [140]. In addition, ceramide VI liposomes were reported to induce G_1_ cell cycle arrest by downregulation of cyclin D1 in osteosarcoma cells [141]. Collectively, our data strongly suggest that cyclin D1 modulation by C5 gel contributes to its enhanced anti-proliferative effects involving suppression of EGFR/ERK signaling.

Matrix metalloproteinase-2 (MMP-2), belonging to the gelatinase subgroup of matrix metalloproteinases, is crucial in various aspects of cancer, including cancer invasion, differentiation, and metastasis by facilitating the proteolysis of structural extracellular matrix proteins [142]. As demonstrated in Table 6, the MMP-2 level was substantially increased in the SEC control group by 4-fold in comparison to the negative control group. This is in accordance with Goździalska et al., reporting MMP-2 overexpression in both nodular and infiltrative basal cell carcinoma [143]. In addition, high MMP-2 expression positively correlated with cancer-associated fibroblasts’ infiltration and was notably associated with lower overall survival rates in skin cutaneous melanoma patients [144].

Conversely, our findings show that treatment of SEC-bearing animals with NIF gel, blank C5 gel, and the optimum C5 gel exhibited invasion-suppressing effects as reflected by the significant decrement of MMP-2 level by 17%, 36.8%, and 57.2%, respectively, relative to the SEC control group (Table 6), where the optimum C5 gel exhibited the greatest effect. This outcome complies with a prior study where NIF treatment produced a dose-dependent reduction in both MMP-2 and MMP-9 activities concomitant with decreased migratory and invasive capacities of cancer cells [21].

Former studies have established that activation of EGFR/ERK signaling pathway promotes tumor invasion by upregulating MMP-2 in multiple cancer types, including esophageal squamous carcinoma, renal clear cell carcinoma, and breast cancer [145,146]. In line with these reports, our results collectively support the involvement of the EGFR/ERK/MMP-2 axis in tumor progression and suggest that the optimum C5 gel-mediated suppression of this pathway contributes to the reduced invasive potential observed in the present study.

Cyclooxygenase-2 (COX-2) is an inducible prostaglandin–endoperoxide synthase-2 enzyme responsible for the production of prostanoids like prostaglandin E_2_ (PGE_2_), which are involved in the modulation of various procarcinogenic effects [147]. COX-2 overexpression has been associated with various oncogenic processes, which include cell proliferation, inhibition of apoptosis, angiogenesis, metastasis, and drug resistance [148]. In the present study, COX-2 levels in the SEC control group were significantly elevated, showing a 5.5-fold increase in comparison to the normal control group (Table 7). This observation agreed with previous reports of COX-2 overexpression across multiple malignancies, including skin cancers such as cutaneous squamous cell carcinoma and melanoma [148].

In contrast, Table 7 showed that treating SEC-bearing mice with NIF gel, blank C5 gel, and the optimum C5 gel resulted in a marked reduction in COX-2 level by 22.3%, 47.8%, and 59.4%, in comparison to the SEC control group, which is consistent with their reported molecular actions. NIF, being fenmate NSAID, is well recognized for its ability to inhibit COX-2 enzymatic activity, resulting in reduced synthesis of pro-inflammatory prostaglandins such as PGE_2_ [149]. In parallel, ceramide VI has been shown to exert anti-tumor effects, including promotion of EGFR/HER receptor degradation and inhibition of downstream proliferative signaling such as ERK activation [126].

Several lines of evidence report that EGFR/ERK signaling pathway serves as a crucial upstream regulator of COX-2 expression, where sustained activation of ERK1/2 enhanced transcriptional activity of pro-inflammatory transcription factors, which include NF-κB, leading to upregulation of COX-2, fostering an environment favorable to cancer progression and inflammation [150]. Additionally, ceramide has been reported to inhibit NF-κB activation independently of EGFR by interfering with upstream signaling pathways such as PKC-mediated translocation, supporting a potential anti-inflammatory role in cancer models [151]. Accordingly, in the current work, the pronounced reduction in COX-2 level observed in the optimum C5 gel group likely reflects complementary suppression of both inflammatory and EGFR-dependent signaling pathways.

#### 3.10.4. Effect on Tumor Caspase-3, MDA Levels, and Total Antioxidant Capacity (TAC)

Caspase-3 is a central executioner caspase in the intrinsic apoptotic pathway, serving as a key effector responsible for the proteolytic cleavage of critical cellular substrates during programmed cell death [152]. As illustrated in Table 7, inoculation of EAC cells in mice led to a notable suppression of apoptosis, evidenced by a significant decrement of the pro-apoptotic activator caspase-3 level by 83.4% in comparison with the normal control. Conversely, caspase-3 level was substantially restored and markedly increased in NIF gel, blank C5 gel, and the optimum C5 gel-treated mice by 5.1, 9.9, and 18.5-fold, respectively, in comparison to the SEC control group (Table 7).

The above finding aligns with a previous study demonstrating that NIF-induced apoptosis in human lung cancer cells through the activation of caspase-3, caspase-9, and caspase-3-mediated PARP cleavage [20]. Consistently, ceramide VI has been shown to promote apoptosis in glioblastoma cells, accompanied by elevated caspase-3 levels [153]. Importantly, these effects might be attributed to the downregulation of oncogenic miR-21-5p, which has been shown to suppress caspase-3 expression in cancer cells [154], indicating that modulation of miR-21 contributes to the restoration of apoptotic signaling observed in the optimum C5 gel.

Oxidative stress resulting from excessive reactive oxygen species (ROS) production that damages cellular macromolecules is a pivotal contributor to tumorigenesis [155]. In the current work, evidence of conspicuous oxidative stress and lipid peroxidation was demonstrated by a marked increment in the MDA level by 6.1-folds, together with a significant reduction in TAC by 78.7% in SEC control group, relative to the normal control group (Table 7). These findings comply with prior reports of elevated MDA levels and impaired antioxidant defenses in non-melanoma skin cancer [156] and breast cancer [157], reflecting both increased ROS generation and compromised antioxidant defenses in tumor cells.

Notably, elevated ROS levels have been reported to modulate EGFR signaling, promoting tumor progression and therapeutic resistance, providing a link between elevated oxidative stress and regulation of EGFR pathway in cancer models [158]. Moreover, increased ROS has been shown to promote pro-apoptotic signaling via caspase-3 activity modulation and alter the balance of BCL-2 family proteins towards apoptosis, thereby linking oxidative stress to both inflammatory and apoptotic pathways in cancer cells [155].

Meanwhile, NIF gel, blank C5 gel, and the optimum C5 gel treatments effectively counteracted these oxidative alterations, evidenced by the significant decline of MDA level by 25.6%, 42.9%, and 63.6%, respectively, coupled with a marked increase in TAC by 1.9, 2.7, and 3.5-fold relative to SEC control group (Table 7). Consistent with our findings, NIF has been reported to alleviate oxido-nitrosative stress in an experimental stavudine-induced neuropathic pain model, supporting its capacity to modulate oxidative stress in vivo [159].

Collectively, the findings demonstrated that the co-incorporation of ceramide VI with NIF in the form of NIF-loaded PEG-CERs resulted in an augmented therapeutic effect and enhanced the attenuation of tumor-associated oxidative stress. Importantly, the antitumor effects of the optimum C5 gel appear to involve complementary modulation of apoptotic, inflammatory, and oxidative-stress pathways rather than an indiscriminate ROS-mediated cytotoxic mechanism. C5 treatment modulated EGFR/ERK1/2 and miR-21-5p expression, accompanied by reduced BCL-2 and increased cleaved Caspase-3 immunoreactivity, while simultaneously reducing COX-2 and MDA and increasing TAC. Thus, the optimized formulation may combine enhanced apoptotic signaling with attenuation of tumor-associated oxidative and inflammatory stress, collectively contributing to its antitumor activity.

#### 3.10.5. In Vivo Histopathological Study

**Figure 11:**
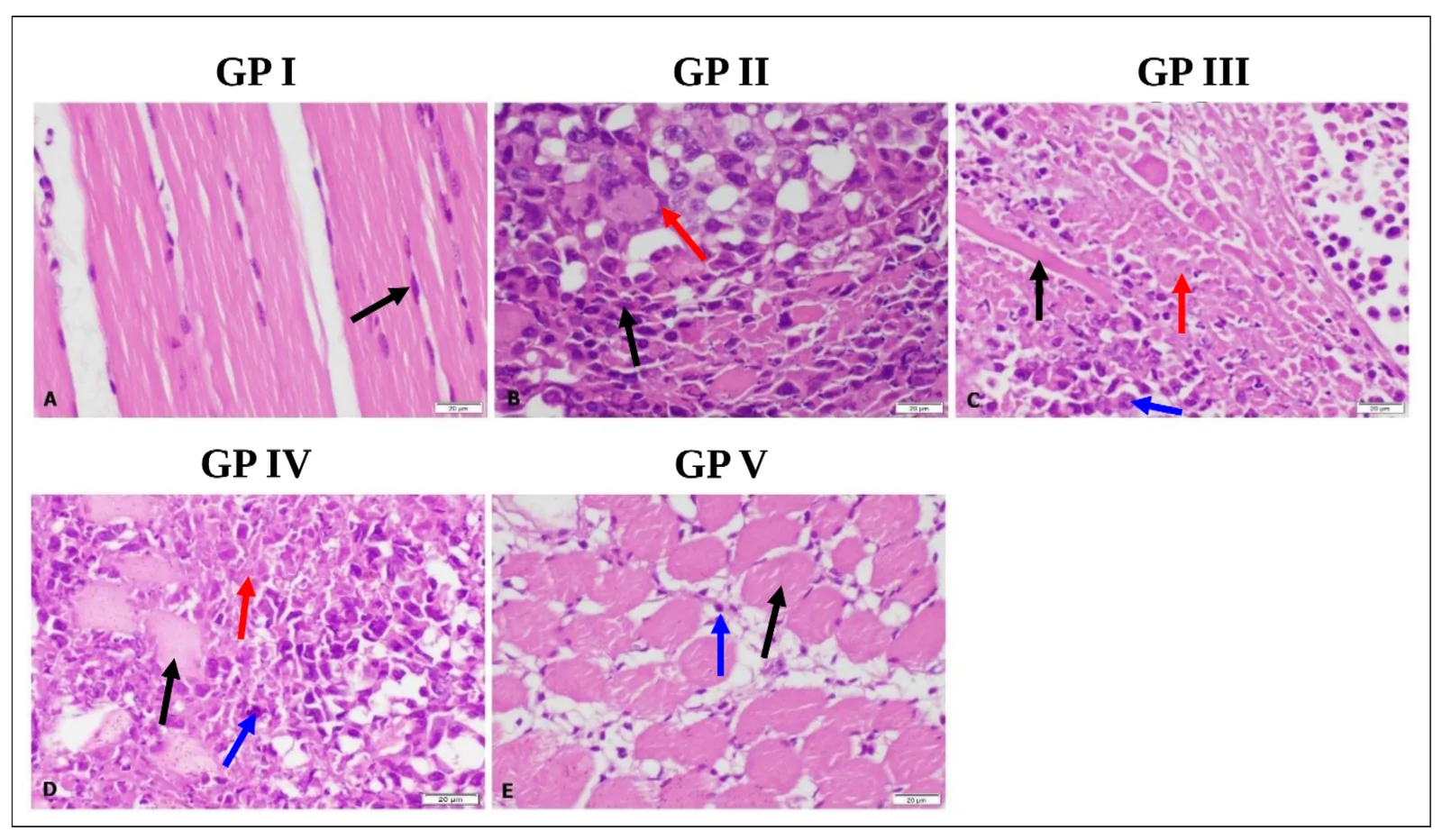
Representative images of tumor tissues of animal groups receiving different treatments at the end of the in vivo study. Histopathological examinations of Group I (**A**) revealed normal histological structure of muscle bundles. Group II (**B**) showed vast clusters of neoplastic cells (black arrow) and giant tumor cells (red arrow). Group III (**C**) demonstrated areas of necrosis within (black arrow), neoplastic cells (red arrow), and mononuclear inflammatory cells infiltration (blue arrow). Group IV (**D**) showed degenerated neoplastic cells (black arrow), necrosed tissue (red arrow), and inflammatory cells infiltration (blue arrow). Concerning Group V (**E**), the examined sections had remarkable regression of infiltration by the malignant cells with a histological structure of muscle bundles similar to Group I (black arrow) and mild inflammation (blue arrow). Hematoxylin and eosin stain ×200.

Histopathological examination was conducted at the end of the in vivo study to confirm the induction of Ehrlich carcinoma and to assess the antitumor effects of the different treatments in comparison to the untreated group. Representative photomicrographs of tumor tissues from all the treated groups and from the SEC control group are illustrated in Figure 11. Microscopic examination of the negative control group (Group I) demonstrated normal histological structure of muscle bundles (Figure 11A). In contrast, the SEC control group (Group II) exhibited extensive clusters of neoplastic cells characterized by nuclear chromachia, high nucleus-to-cytoplasm ratio, and marked anisocytosis with the presence of tumor giant cells, consistent with previous reports [160] (Figure 11B).

Both the NIF gel-treated group (Group III) (Figure 11C) and the blank C5 gel-treated group (Group IV) (Figure 11D) revealed mild and moderate improvements of histopathological changes, confirmed by the existence of areas of necrosis within the neoplastic cells and mononuclear inflammatory cells infiltration. On the other hand, the optimum C5 gel (Group V) (Figure 11E) showed marked improvement, characterized by a significant reduction in neoplastic cells, extensive areas of necrosis, and a relatively small subcutaneous tumor mass containing abundant degenerated and necrotic tumor cell debris. This ensured the antitumor effect supremacy of the optimum C5 gel, compared to other treatment groups.

As previously mentioned in the permeation section, ceramides integrated into the phospholipid membrane can result in transbilayer lipid movement, vesicle budding, and membrane fusion [68,110,113]. Furthermore, the “flip-flop” diffusion of ceramide across lipid bilayers may disrupt the membrane permeability barrier [113] and thereby enhancing the tumor accumulation of the blank C5 gel and the optimum C5 gel compared with the pure NIF.

#### 3.10.6. Immunohistochemical Studies of B-Cell LYMPHOMA 2 (BCL-2)

**Figure 12:**
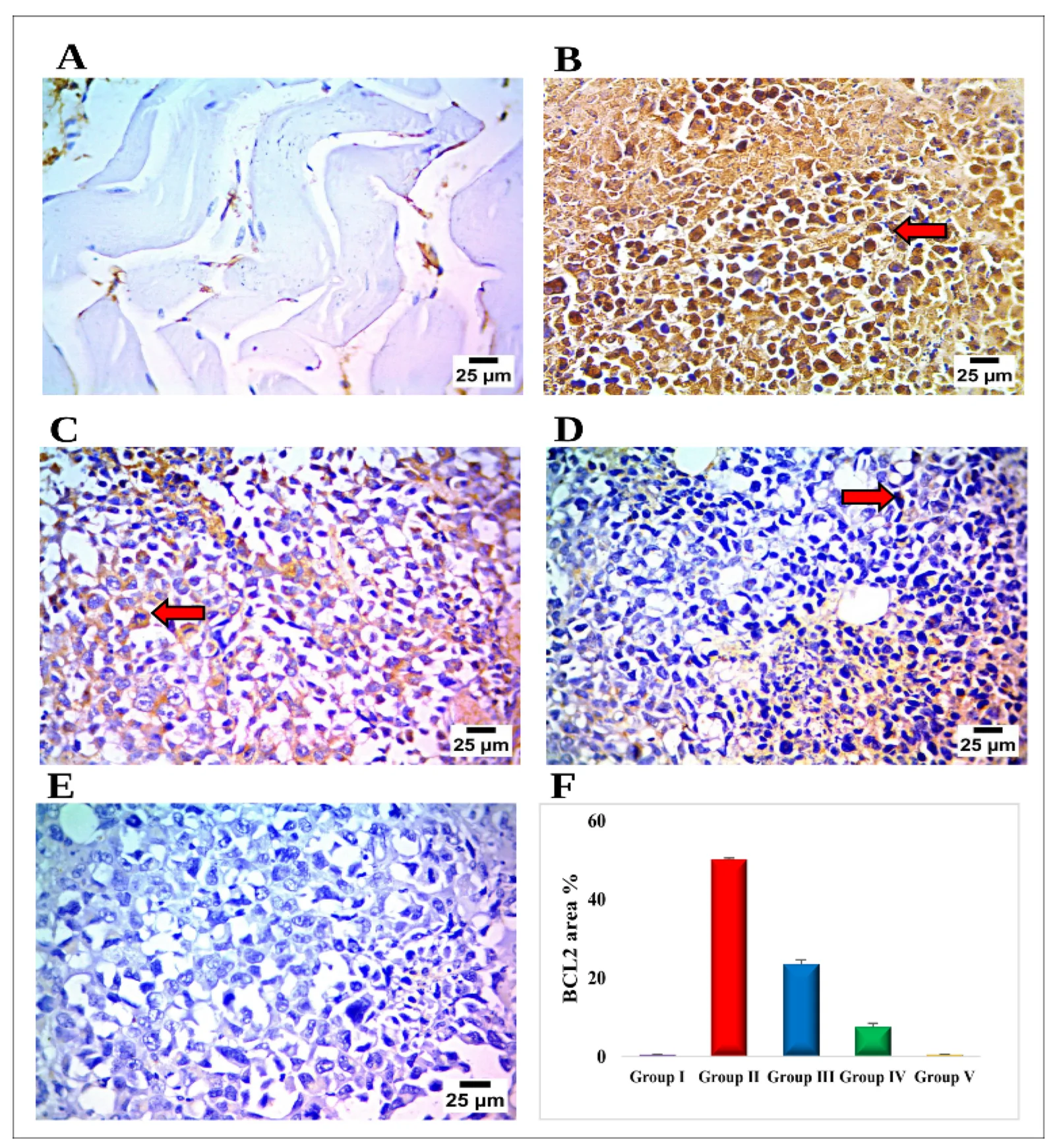
Representative photomicrographs of the immunohistochemical determination of BCL-2 expression in tumor tissue across experimental groups. The brown color in the presented figure indicates the immunostaining of BCL-2 as follows: (**A**) Group I: exhibiting normal, limited to negative BCL-2 expression in muscle fibers. (**B**) Group II showing intense, severe positive expression for BCL-2 in neoplastic cells (arrow). (**C**) Group III showing moderate positive BCL-2 expression in neoplastic cells (arrow). (**D**) Group IV showing mild positive expression for BCL-2 in some neoplastic cells (arrow). (**E**) Group V showing negative BCL-2 expression. (**F**) The chart represents the quantification of the BCL-2 expression area percentage. Data are presented as mean ± SEM. Significant difference is considered at *p* < 0.05.

B-Cell Lymphoma 2 (BCL-2) is a key anti-apoptotic protein that inhibits programmed cell death by binding pro-apoptotic molecules like Bax and Bak, thereby blocking mitochondrial apoptosis pathways and caspase activation [161]. Its overexpression is associated with enhanced tumor progression; therefore, BCL-2 serves as an important marker of anti-apoptotic activity and tumor cell survival that can be assessed reliably by immunohistochemistry. Immunohistochemical analysis revealed that BCL-2 was strongly expressed positively by SEC control group (Figure 12B) compared to the normal control group (Figure 12A), reflecting the tumor’s anti-apoptotic adaptation. Quantitative image analysis confirmed this marked difference, with BCL-2-positive reaction area reaching 50.17 ± 0.36 in the SEC control compared with 0.49 ± 0.11 in the normal control (Figure 12F). This finding aligns with those of Dahran et al., who had also found a marked positive expression of BCL-2 in neoplastic cells of Ehrlich carcinoma [162].

On the other hand, both treated groups with NIF gel and blank C5 gel (Figure 12C,D) showed a notable reduction in BCL-2 expression compared to the SEC control group with positive reaction areas of 23.48 ± 1.10 and 7.58 ± 0.83, respectively. Notably, optimum C5 gel treatment (Figure 12E) showed the least value of BCL-2 positive staining among all treatments, with expression levels approaching those of the normal control group (0.51± 0.1), indicating re-establishment of physiological apoptotic balance. This effect might be attributed to miRNA-21-5p downregulation as demonstrated by RT-PCR analysis in the present study. These findings are in harmony with previous reports indicating that oncogenic signaling pathways and microRNAs, particularly miR-21-5p, can directly upregulate BCL-2 expression, thereby promoting apoptosis resistance and chemoresistance in cancer cells [163].

Collectively, modulation of BCL-2 expression appears to be a key contributor to the enhanced apoptotic response and antitumor efficacy of the optimum C5 gel formulation. To the best of our knowledge, this study provides the first evidence that such effects are mediated, at least in part, through miR-21-5p downregulation, revealing a novel mechanistic link between microRNA modulation and apoptosis induction underlying its anticancer activity.

### 3.11. Study Limitations

Despite these findings, some limitations should be acknowledged. Although the subcutaneous solid Ehrlich carcinoma (SEC) model is a well-established and reproducible model for evaluating solid-tumor growth and antitumor responses, it does not fully recapitulate the cellular origin, tissue architecture, or molecular characteristics of primary human cutaneous malignancies. Therefore, the present findings should be considered proof-of-concept evidence of the in vivo antitumor activity of the NIF-loaded PEG-CERs gel rather than a direct prediction of its clinical efficacy against human skin cancer. Further validation in disease-specific cutaneous cancer models, including chemically induced, syngeneic, or xenograft models of epidermal carcinoma or melanoma, is warranted to establish the translational relevance of these findings.

A further limitation is the absence of a conventional standard-of-care anticancer comparator in the in vivo study. Although the 62.8% TIR demonstrates substantial antitumor activity within the present experimental model, direct comparison with published SEC studies is limited by differences in treatment dose, route, duration, and experimental design. Thus, equivalence or superiority to established chemotherapy cannot be concluded. Future studies incorporating an appropriate standard anticancer comparator under identical experimental conditions would help define the relative efficacy and therapeutic advantage of the optimized formulation.

## 4. Conclusions

In this study, Niflumic acid (NIF) was successfully repurposed and encapsulated into PEGylated cerosomes (PEG-CERs) as an effective topical nanoplatform for solid epithelial carcinoma management. Statistical optimization identified the non-sonicated formulation C5 (composed of bioactive Ceramide VI, L-α-phosphatidylcholine, and Brij 93) as the optimal system, demonstrating an elongated tubular morphology, high entrapment efficiency (96.71 ± 0.0%), nanoscale size (292.95 ± 0.78 nm), high negative zeta potential (−37.5 ± 0.57 mV), and confirmed drug amorphization and physicochemical compatibility by DSC and FTIR analyses. The formulated C5 gel exhibited a 2.02-fold enhancement in ex vivo skin permeation flux compared to pure NIF gel, without interference from endogenous tissue components. In vivo evaluation in SEC-bearing mice validated the augmented antitumor efficacy of the C5 gel, achieving an 80% survival rate, marked tumor volume reduction (526.8 ± 41.7 mm^3^), and a superior tumor inhibition rate of 62.8%. Mechanistically, this therapeutic response was driven by significant transcriptional downregulation of EGFR (47.9%), ERK1 (58.2%), ERK2 (73.0%), and miR-21-5p (67.3%), alongside marked reductions in Cyclin D1 (61.8%), MMP-2 (57.2%), and COX-2 (59.4%) levels. Furthermore, the C5 gel restored physiological apoptotic balance—evidenced by an 18.5-fold elevation in Caspase-3 and near-complete normalization of BCL-2 immunostaining, while concurrently resolving tumor-associated oxidative inflammation via a 63.6% reduction in MDA and a 3.5-fold increase in TAC. Consequently, NIF-loaded PEG-CERs represent a promising, synergistic topical nanoplatform combining targeted MAPK/ERK and miR-21 pathway modulation with anti-inflammatory activity for skin cancer management.

## Figures and Tables

**Table 1:** 2^3^.3^1^ full factorial design and desirability constraints for the preparation and optimization of NIF-loaded PEG-CERs.

Factors (Independent Variables)	Levels		
	Low	Medium	High
X1: Ceramide Type	Ceramide VI	Ceramide III	Ceramide IIIB
X2: Ceramide Amount	5 mg		10 mg
X3: Brij Type	Brij 35		Brij 93
X4: Sonication Time	0		10 min
Responses (Dependent variables)	Desirability Constraints		
Y1: EE%	Maximize		
Y2:VS	Minimize		
Y3: PDI	In range		
Y4: ZP	In range		

EE%, encapsulation efficiency percent; VS, vesicle size; PDI, polydispersity index; ZP, zeta potential.

**Table 2:** Experimental runs, independent variables, and measured response of the experimental design of NIF-loaded PEG-CERs.

Formulation Code of NIF-Loaded PEG-CERs	Factors				Responses			
	Ceramide Type (X1)	Ceramide Amount (mg) (X2)	Brij Type (X3)	Sonication Time (min) (X4)	Y1: EE % (Mean ± SD)	Y2: VS (nm) (Mean ± SD)	Y3: PDI (Mean ± SD)	Y4: ZP (mV) (Mean ± SD)
**C1**	Ceramide VI	5	Brij 93	0	94.64 ± 0.36	309.85 ± 3.18	0.49 ± 0.0	−39.3 ± 0.28
**C2**	Ceramide VI	5	Brij 93	10	74.24 ± 1.65	89.62 ± 0.22	0.37 ± 0.01	−28 ± 0.0
**C3**	Ceramide VI	5	Brij 35	0	91.98 ± 1.69	243 ± 0.57	0.39 ± 0.0	−34.1 ± 0.99
**C4**	Ceramide VI	5	Brij 35	10	71.54 ± 0.75	79.02 ± 0.58	0.23 ± 0.0	−22.55 ± 0.64
**C5**	Ceramide VI	10	Brij 93	0	96.71 ± 0	292.95 ± 0.78	0.47 ± 0.0	−37.5 ± 0.57
**C6**	Ceramide VI	10	Brij 93	10	77.55 ± 0	91.19 ± 0.25	0.37 ± 0.0	−29.65 ± 0.92
**C7**	Ceramide VI	10	Brij 35	0	93.19 ± 0.35	266.9 ± 0.42	0.44 ± 0.0	−33.9 ± 0.71
**C8**	Ceramide VI	10	Brij 35	10	72.55 ± 0.47	87.81 ± 0.22	0.26 ± 0.0	−24.95 ± 2.19
**C9**	Ceramide III	5	Brij 93	0	94.40 ± 0.24	242.8 ± 0	0.43 ± 0.01	−34.85 ± 0.64
**C10**	Ceramide III	5	Brij 93	10	72.56 ± 0	96.86 ± 0.07	0.38 ± 0.0	−33.5 ± 0.71
**C11**	Ceramide III	5	Brij 35	0	92.26 ± 0	243.2 ± 0.71	0.46 ± 0.07	−30.2 ± 0.28
**C12**	Ceramide III	5	Brij 35	10	67.71 ± 1.42	86.39 ± 0.69	0.24 ± 0.0	−31 ± 1.41
**C13**	Ceramide III	10	Brij 93	0	94.49 ± 0	257.15 ± 0.49	0.45 ± 0.01	−35.35 ± 0.35
**C14**	Ceramide III	10	Brij 93	10	70.09 ± 0	97.6 ± 0	0.38 ± 0.01	−30.55 ± 0.07
**C15**	Ceramide III	10	Brij 35	0	93.36 ± 0.81	274.8 ± 0.71	0.45 ± 0.01	−33.45 ± 0.07
**C16**	Ceramide III	10	Brij 35	10	70.08 ± 0.81	100.6 ± 1.41	0.35 ± 0.0	−30.45 ± 1.77
**C17**	Ceramide IIIB	5	Brij 93	0	90.21 ± 0.28	270.1 ± 1.13	0.45 ± 0.01	−32.3 ± 0.0
**C18**	Ceramide IIIB	5	Brij 93	10	71.15 ± 0	83.86 ± 0.06	0.29 ± 0.0	−31 ± 0.57
**C19**	Ceramide IIIB	5	Brij 35	0	92.89 ± 0.16	254.1 ± 1.27	0.43 ± 0.0	−34.55 ± 0.35
**C20**	Ceramide IIIB	5	Brij 35	10	70.51 ± 0.51	86.79 ± 0.37	0.27 ± 0.0	−23.35 ± 1.63
**C21**	Ceramide IIIB	10	Brij 93	0	93.52 ± 0.011	372.4 ± 0.14	0.49 ± 0.01	−34.9 ± 0.28
**C22**	Ceramide IIIB	10	Brij 93	10	73.79 ± 0.83	86.51 ± 0.16	0.28 ± 0.0	−21.4 ± 0.85
**C23**	Ceramide IIIB	10	Brij 35	0	91.68 ± 0	251.2 ± 0.99	0.45 ± 0.0	−34.4 ± 0.0
**C24**	Ceramide IIIB	10	Brij 35	10	75.17 ± 0.07	88.84 ± 0.57	0.25 ± 0.0	−21.45 ± 0.35

All the prepared NIF-loaded PEG-CERs contained the same amount of NIF (10 mg), Brij 35 or Brij 93 (5 mg) along with phosphatidylcholine (100 mg). EE%, encapsulation efficiency percent; VS, vesicle size; PDI, polydispersity index; ZP, zeta potential. Data are presented as mean ± SD (n = 3).

**Table 3:** Primers used in RT-PCR.

Gene	Primer Sequence
EGFR	F: 5′-TCT TCA AGG ATG TGA AGT GTG-3′ R: 5′-TGT ACG CTT TCG AAC AAT GT-3′
ERK 1	F: 5′-TGGCTTTCTGACGGAGTATG-3′ R: 5′-GGTCCAGGTAGTGCTTGC-3′
ERK 2	F: 5′-CCTCAAGCCTTCCAACCTC-3′ R: 5′-GCCCACAGACCAAATATCAATG-3′
miR-21-5p	F: 5′-GCCGCTAGCTTATCAGACTG-3′ R: 5′-GTGCAGGGTCCGAGGT-3′
GAPDH	F: 5′-CAAGGTCATCCATGACAACTTTG-3′ R: 5′-GTCCACCACCCTGTTGCTGTAG-3′

**Table 4:** Factorial analysis outcomes of PEG-CERs with the predicted and observed outcomes of the optimal NIF-loaded PEG-CERs formulation (C5).

Responses	R^2^	Adjusted R^2^	Predicted R^2^	Adequate Precision	Significant Factors
Y1: EE% Y2: VS (nm) Y3: PDI Y4: ZP (mV)	0.98 0.95 0.85 0.69	0.97 0.94 0.83 0.66	0.97 0.93 0.80 0.60	43.24 27.44 19.23 13.46	X1, X2, X3, X4 X2, X3, X4 X3, X4 X1, X3, X4
Responses	Y1: EE%	Y2: VS (nm)	Y3: PDI	Y4: ZP (mV)	
Predicted values of the selected formula (C5)	96.17	291.46	0.48	−36	
Observed values of selected formula (C5)	96.71	292.95	0.47	−37.5	

EE%, encapsulation efficiency percent; VS, vesicle size; PDI, polydispersity index; ZP, zeta potential.

**Table 5:** The effect of storage on the physical characteristics of the optimal NIF-loaded PEG-CERs formulation (C5).

Parameter	C5 (Fresh)	C5 (After 45 Days)	C5 (After 90 Days)
EE%	96.71 ± 0	95.66 ± 0.48	95.71 ± 0.41
VS (nm)	292.95 ± 0.78	287.8 ± 4.24	285.1 ± 2.83
PDI	0.47 ± 0.0	0.46 ± 0.02	0.45 ± 0.01
ZP (mV)	−37.5 ± 0.57	−36.1 ± 0.57	−37.4 ± 0.57

EE%, encapsulation efficiency percent; VS, vesicle size; PDI, polydispersity index; ZP, zeta potential.

**Table 6:** Effect of the optimum C5 gel treatment of SEC-bearing mice on tumor Cyclin D1, MMP2, and COX-2 levels.

No.	Group	Cyclin D1 (pg/mg Protein)	MMP-2 (pg/mg Protein)	COX-2 (ng/mg Protein)
I	Normal control	305.5 ± 17.09	253.7 ± 9.11	13.05 ± 0.545
II	SEC control	1226 ± 20.45 ^a^	1023 ± 45.03 ^a^	71.69 ± 1.026 ^a^
III	SEC bearing mice + NIF gel	898.3 ± 17.33 ^a,b^	848.8 ± 13.49 ^a,b^	52.55 ± 0.504 ^a,b^
IV	SEC bearing mice + Blank C5 gel	693.5 ± 22.84 ^a,b,c^	646.6 ± 13.16 ^a,b,c^	40.41 ± 0.822 ^a,b,c^
V	SEC bearing mice + optimum C5 gel	468.8 ± 14.17 ^a,b,c,d^	437.7 ± 11.72 ^a,b,c,d^	29.11 ± 0.306 ^a,b,c,d^

Cyclin D1, MMP2, and COX-2 protein levels are expressed as mean ± SEM. The data were analyzed using one-way ANOVA followed by Tukey’s multiple comparisons test. The difference with *p* < 0.05 was considered statistically significant. ^a^ statistically significant from normal controls (Group I), ^b^ statistically significant from SEC control (Group II), ^c^ statistically significant from SEC bearing mice receiving NIF gel (Group III), ^d^ statistically significant from SEC-bearing mice receiving blank C5 gel (Group IV), MMP-2: matrix metalloproteinase-2, COX-2: cyclooxygenase-2, SEC: Solid Ehrlich carcinoma, NIF: niflumic acid.

**Table 7:** Effect of optimum NIF-loaded PEG-CERs gel treatment of SEC-bearing mice on tumor Caspase-3, MDA, and TAC levels.

No.	Group	Caspase-3 (ng/mg Protein)	MDA (nmol/g Tissue)	TAC (mmol/g Protein)
**I**	Normal control	1.281 ± 0.046	0.788 ± 0.017	1.82 ± 0.097
**II**	SEC control	0.212 ± 0.021 ^a^	4.797 ± 0.122 ^a^	0.387 ± 0.018 ^a^
**III**	SEC bearing mice + NIF gel	1.075 ± 0.047 ^b^	3.57 ± 0.158 ^a,b^	0.756 ± 0.019 ^a,b^
**IV**	SEC bearing mice + Blank C5 gel	2.116 ± 0.147 ^a,b,c^	2.74 ± 0.156 ^a,b,c^	1.038 ± 0.065 ^a,b,c^
**V**	SEC bearing mice + optimum C5 gel	3.928 ± 0.178 ^a,b,c,d^	1.745 ± 0.109 ^a,b,c,d^	1.366 ± 0.073 ^a,b,c,d^

Caspase-3 protein level, MDA, and TAC levels are expressed as mean ± SEM. Caspase-3 was measured using ELISA, while MDA and TAC were determined spectrophotometrically. The data were analyzed using one-way ANOVA followed by Tukey’s multiple comparisons test. A difference with *p* < 0.05 was considered to be statistically significant. ^a^ statistically significant from normal controls (Group I), ^b^ statistically significant from SEC control (Group II), ^c^ statistically significant from SEC bearing mice receiving NIF gel (Group III), ^d^ statistically significant from SEC bearing mice receiving blank C5 gel (Group IV), MDA: malondialdehyde, TAC: total antioxidant capacity, SEC: Solid Ehrlich carcinoma, NIF: niflumic acid.

## Data Availability

The original contributions presented in this study are included in the article. Further inquiries can be directed to the corresponding authors.

## Abbreviations

The following abbreviations are used in this manuscript:

ABC	Avidin-biotin-peroxidase complex
ANOVA	Analysis of variance
BCL-2	B-Cell Lymphoma 2
BCS	Biopharmaceutical Classification System
CERs	Cerosomes
COX-2	Cyclooxygenase-2
DAB	Diaminobenzidine
DMPC	Dimyristoylphosphatidylcholine
DSC	Differential Scanning Calorimetry
EAC	Ehrlich Ascites Carcinoma
EE%	Entrapment efficiency percentage
EGFR	Epidermal growth factor receptor
ELISA	Enzyme-linked immunosorbent assay
ERK	Extracellular signal-regulated kinase
ERK1	Extracellular signal-regulated kinase 1
ERK2	Extracellular signal-regulated kinase 2
H&E	Hematoxylin and eosin
HLB	Hydrophilic–lipophilic balance
HPMC	Hydroxypropyl methylcellulose
Jak-3	Janus Kinase-3
Jss	Steady state flux
MAPK	Mitogen-activated protein kinase
MDA	Malondialdehyde
miR-21	MicroRNA-21
MMP-2	Matrix metalloproteinase-2
MMP-9	Matrix metalloproteinase-9
NF-κB	Nuclear factor kappa B
NIF	Niflumic acid
NSAIDs	Non-steroidal anti-inflammatory drugs
NSCLC	Non-small cell lung carcinoma
Papp	Permeability coefficient
PBS	Phosphate-buffered saline
PDI	Polydispersity index
PEG-CERs	PEGylated cerosomes
PGE2	Prostaglandin E2
POPC	1-palmitoyl-2-oleoyl-sn-glycero-3-phosphocholine
POPE	1-palmitoyl- 2-oleoyl-sn-glycero-3-phosphoethanolamine
PPAR-α	Peroxisome proliferator-activated receptor alpha
Q	Cumulative quantity of the drug permeated
ROS	Reactive oxygen species
RT-PCR	Real-Time Polymerase Chain Reaction
SEC	Solid Ehrlich carcinoma
Spry2	Sprouty2
STAT-3	Signal transducer and activator of transcription-3
TAC	Total antioxidant capacity
TEM	Transmission electron microscopy
TIR	Tumor inhibition rate
TPP	Tripolyphosphate
UV	Ultraviolet
VHL	Von Hippel–Lindau
VS	Vesicle size
ZP	Zeta potential
